# X-Ray Diffraction Line Broadening: Modeling and Applications to High-T_c_ Superconductors

**DOI:** 10.6028/jres.098.026

**Published:** 1993

**Authors:** Davor Balzar

**Affiliations:** National Institute of Standards and Technology, Boulder, CO 80303-3328

**Keywords:** diffraction line broadening, lattice defects, profile fitting, superconductors, Voigt function, Warren-Averbach analysis, x-ray diffraction

## Abstract

A method to analyze powder-diffraction line broadening is proposed and applied to some novel high-*T*_c_ superconductors. Assuming that both size-broadened and strain-broadened profiles of the pure-specimen profile are described with a Voigt function, it is shown that the analysis of Fourier coefficients leads to the Warren-Averbach method of separation of size and strain contributions. The analysis of size coefficients shows that the “hook” effect occurs when the Cauchy content of the size-broadened profile is underestimated. The ratio of volume-weighted and surface-weighted domain sizes can change from ~1.31 for the minimum allowed Cauchy content to 2 when the size-broadened profile is given solely by a Cauchy function. If the distortion co-efficient is approximated by a harmonic term, mean-square strains decrease linearly with the increase of the averaging distance. The local strain is finite only in the case of pure-Gauss strain broadening because strains are then independent of averaging distance. Errors of root-mean-square strains as well as domain sizes were evaluated. The method was applied to two cubic structures with average volume-weighted domain sizes up to 3600 Å, as well as to tetragonal and orthorhombic (La-Sr)_2_CuO_4_, which exhibit weak line broadenings and highly overlapping reflections. Comparison with the integral-breadth methods is given. Reliability of the method is discussed in the case of a cluster of the overlapping peaks. The analysis of La_2_CuO_4_ and La_1.85_M_0.15_CuO_4_(M = Ca, Ba, Sr) high-*T*_c_ superconductors showed that microstrains and incoherently diffracting domain sizes are highly anisotropic. In the superconductors, stacking-fault probability increases with increasing *T*_c_; microstrain decreases. In La_2_CuO_4_, different broadening of (*h*00) and (0*k*0) reflections is not caused by stacking faults; it might arise from lower crystallographic symmetiy. The analysis of Bi-Cu-O superconductors showed much higher strains in the [001] direction than in the basal *a-b* plane. This may be caused by stacking disorder along the *c*-axis, because of the two-dimensional weakly bonded BiO double layers. Results for the specimen containing two related high-*T*_c_ phases indicate a possible mechanism for the phase transformation by the growth of faulted regions of the major phase.

## 1. Introduction

X-ray diffraction is one of the oldest tools used to study the structure of matter. In 1912, Laue [1] demonstrated in a single experiment that crystals consist of regularly repeating elementary building blocks, and that x rays show wave nature. Since then, x-ray diffraction has become one of the basic and the most widely used methods for characterization of a broad range of materials.

### 1.1 Powder X-Ray Diffraction

Many materials are not available in a monocrystal form. Moreover, powders and bulk materials are more easily obtainable, practical, and less expensive. A powder-diffraction experiment requires an order-of-magnitude shorter time than a monocrystal experiment. Thus, powder diffraction is used very often. However, because data are of lower quality and peaks are generally highly overlapped at higher diffracting angles, until 25 years ago powder diffraction was mostly used for qualitative phase analysis. Through advances by Rietveld [2, 3], powder-diffraction patterns become used in structure analysis, so-called structure (Rietveld) refinement. Development of fast on-line computer-controlled data acquisition has allowed a quick analysis of the whole diffraction pattern. Table 1 summarizes uses of different diffraction line-profile parameters in various types of analyses (after Howard and Preston [4]). We shall focus on line-profile analysis to obtain information about microstructural properties of materials: microstrains in the lattice and size of incoherently diffracting domains in crystals.

### 1.2 Diffraction-Line Broadening

Diffraction from crystal planes occurs at well-defined angles that satisfy the Bragg equation

$$\lambda =2d_{hkl}\sin\theta_{hkl}.$$ (1)

Theoretically, intensity diffracted from an infinite crystal should consist of diffraction lines without width (Dirac delta functions) at some discrete diffraction angles. However, both instrument and specimen broaden the diffraction lines, and the observed line profile is a convolution of three functions [5, 6]

h(2θ)=[(ω∗γ)∗f](2θ)+background. (2)

Wavelength distribution and geometrical aberrations are usually treated as characteristic for the particular instrument (instrumental profile):

g(2θ)=(ω∗γ)(2θ). (3)

To obtain a specimen’s microstructural parameters, the specimen (physically) broadened profile *f* must be extracted from the observed profile *h*.

Origins of specimen broadening are numerous. Generally, any lattice imperfection will cause additional diffraction-line broadening. Therefore, dislocations, vacancies, interstitials, substitutions, and similar defects manifest themselves through the lattice strain. If a crystal is broken into smaller incoherently diffracting domains by dislocation arrays, stacking faults, twins, or any other extended imperfections, then domain-size broadening occurs.

### 1.3 Superconductivity and Defects

Since discovery of the ~90 K superconductor YBa_2_Cu_3_O_7−_*_δ_* [7], it became clear that the novel superconductivity relates closely to defects in structure. Both point and extended defects relate closely to the physical properties of superconductors [8, 9]. Defects play an important role both in the critical superconducting transition temperature *T*_c_ [10] and in the critical current density *J*_c_ [H]. Some theories also connect *T*_c_ with lattice distortion [12], with strains around dislocations [13], and with interaction of current carriers and the elastic-strain field [14].

The *T*_c_ of YBa_2_Cu_3_O_7−_*_δ_*, for instance, depends strongly on the oxygen stoichiometry, that is, number of oxygen vacancies in the charge-reservoir layers and their arrangement (see Jorgensen [15] and references therein). Superconductivity in La_2_CuO_4_ appears either by the partial substitution of La with Sr, Ba, Ca [16, 17], or by the introduction of interstitial oxygen defects in the La_2_O_2_ layer [18]. Some substitutions, especially on Cu sites, destroy the superconductivity.

For classical superconductors, *J*_c_ can be drastically increased by introducing defects to pin magnetic flux vortices. The layered structure of high-*T*_c_ cuprates causes the vortices to be pinned in the form of pancakes, rather than long qflinders [19]. Because of relatively small coherence length of vortices, pinning can not be increased in the classical way by introducing second-phase precipitates. Instead, submicroscopic lattice defects caused by local stoichiometry fluctuations, vacancies, substitutions, Guinier-Preston zones, and the strain field of small coherent precipitates are much more effective. Especially in highly anisotropic Tl-based and Bi-based cuprates, substitutions are very successful. Even a 5% Mg for Ba substitution in Tl_2_Ba_2_CaCu_2_O_8_ increases *J*_c_ by 25% [20].

### 1.4 Purpose of the Study

We know that defects have a very important role in novel high-*T*_c_ superconductivity. Defects can be characterized and quantified by analyzing the x-ray diffraction broadening. Basically, there are two approaches:

- The Stokes deconvolution method [21] combined with the Warren and Averbach analysis [22] give the most rigorous and unbiased approach because no assumption about the analytical form of diffraction-peak shape is required. However, when peaks overlap and specimen broadening is comparable with the instrumental broadening, the Stokes method gives unstable solutions and large errors or can not be performed at all. To obtain reliable results, proper corrections have to account for truncation, background, sampling, and the standard’s errors [23].
- The simplified integral-breadth methods (summarized by Klug and Alexander [24]) are more convenient and easier to use, but they require that size and strain broadening are modeled by either Cauchy or Gauss functions. Experience has shown, however, that in most cases both size and strain profiles can not be satisfactorily represented with either function. However, there is some theoretical and experimental evidence that the effect of small-domain-size broadening produces long profile tails of the Cauchy function, and that the lattice-strain distribution is more Gauss-like. Langford [25, 26] used the convolution of Cauchy and Gauss functions (Voigt function) to model specimen broadening. However, the results obtained by the integral-breadth and Warren-Averbach analyses are usually not comparable; the first methods give volume-weighted domain sizes and upper limit of strain; the second gives surface-weighted domain sizes and mean-square strain averaged over some distance perpendicular to diffracting planes.

Unfortunately, most high-*T*_c_ superconductors show weak peak broadening (because of high annealing temperatures) and strong peak overlapping (because of relatively complicated crystal structures), which makes it very difficult to apply the Stokes deconvolution method to extract pure specimen broadening. The aim in this study is twofold:

- To develop a reliable method for analysis of a pattern with highly overlapping reflections and weak structural broadening, and to compare it with the previously described approaches. It will be shown that the Voigt-function modeling of the specimen broadening concurs with the Warren-Averbach approach.
- To apply the method to the same high-*T*_c_ superconductors and conclude how much information about defects can be extracted from analysis of the x-ray diffraction broadening.

## 2. Previous Studies

### 2.1 Size and Strain Broadening

Some important methods to extract specimen size and strain broadening and information about domain sizes and strains will be reviewed briefly. An excellent review about Fourier methods and integral-breadth methods is given by Klug and Alexander [24]. A survey of single-line methods was authored by Delhez, de Keijser, and Mittemeijer [27]. The use of variance (reduced second moment of the line profile) in the analysis of broadening will not be treated here. Wilson described the contributions to variance by crystallite size [28] and strain [29].

#### 2.1.1 Determination of the Pure Specimen-Broadened Profile

As mentioned in Sec. 1.2, before the specimen’s size and strain broadening can be obtained, the observed profile must be corrected for instrumental broadening. Most used methods are the Fourier-transform deconvolution method [30, 21] and simplified integral-breadth methods that rely on some assumed analytical forms of the peak profiles. The iterative method of successive foldings [31, 32] is not used extensively, and will not be considered here.

##### Deconvolution Method of Stokes

From Eqs. (2) and (3), it follows that deconvolution can be performed easily in terms of Fourier transforms of respective functions:

$$F(n)=\frac{H(n)}{G(n)}.$$ (4)

**Fig. 1:**
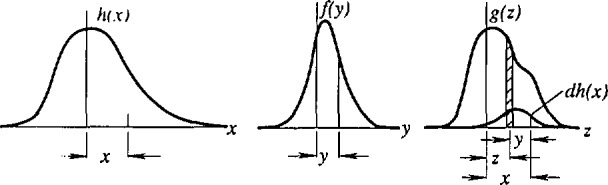
Observed profile n is a convolution of the instrumental profile *g* with the specimen profile *f*. Adapted from Warren [59].

Hence, the physically broadened profile *f* is retrieved from the observed profile *h* without any assumption on the peak-profile shape (see Fig. 1). This method is the most desirable approach because it is totally unbiased. However, because of the deconvolution process, there are many problems. Equation (4) may not give a solution if the Fourier coefficients of the *f* profile do not vanish before those of the *g* profile. Furthermore, if physical broadening is small compared with instrumental broadening, deconvolution becomes too unstable and inaccurate [33, 34]. If the *h* profile is 20% broader than the *g* profile, this gives an upper limit of about 1000 Å for the determination of effective domain size [34]. Regardless of the degree of broadening, deconvolution produces unavoidable profile-tail ripples because of truncation effects. To obtain reliable results, these errors have to be corrected, along with errors of incorrect background, sampling, and the standard specimen [23, 35, 36]. The largest problem, however, is peak overlapping. If the complete peak is not separated, the only possible solution is to try to reconstruct the missing parts. That would require some assumption on the peak-profile shape, that is introduction of bias into the method. The application of the Stokes method is therefore limited to materials having the highest crystallographic symmetry.

##### Integral-Breadth Methods

The basic assumption of these methods is that diffraction profiles can be approximated with some analytical function. In the beginning, two commonly used functions were Gauss

$$I(x)=I(0)\;\exp\;\left[-\pi \frac{x^{2}}{\beta_{G}^{2}}\right]$$ (5)

and Cauchy

$$I(x)=I(0)\frac{1}{\frac{\beta_{C}^{2}}{\pi^{2}}+x^{2}}.$$ (6)

From the convolution integral, it follows easily that

$$\beta_{hC}=\beta_{gC}+\beta_{fC}$$ (7)

for Cauchy profiles, and

$$\beta_{hG}^{2}=\beta_{gG}^{2}+\beta_{fG}^{2}$$ (8)

for Gauss profiles. However, the observed x-ray diffraction line profiles can not be well represented with a simple Cauchy or Gauss function [24, 37]. But they are almost pure Cauchy at highest angles because the dominant cause of broadening becomes the spectral distribution in radiation [24]. Different geometrical aberrations of the instrument are difficult to describe with simple analytical functions. In the case of closely Gaussian broadening of *γ*, following Eq. (3), the instrumental line profile can be best described by a convolution of Cauchy and Gauss functions, which is the Voigt function. Experience shows that the Voigt function [38] (or its approximations, pseudo-Voigt [39, 40] and Pearson-VII [41, 42]) fits very well the observed peak profiles [25, 43, 37]. The Voigt function is usually represented following Langford [25]

I(x)=I(0)(ββC)Re[erfi(π1/2xβG+ik)]. (9)

Here, the complex error function is defined as

$$\text{erfi}(z)=\exp(-z^{2})[1-\text{erf}(-iz)].$$ (10)

**Fig. 2:**
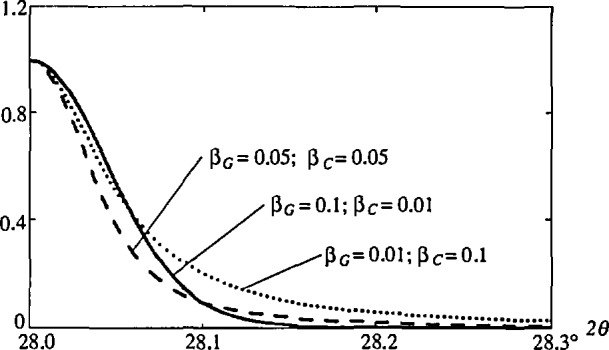
Voigt functions for different values of Cauchy and Gauss integral breadths. Adapted from Howard and Preston [4].

Its evaluation can be accomplished using Sundius. [44] or Armstrong [45] algorithms with eight-digit accuracy. Useful information about the Voigt function can be found in papers of Kielkopf [46], Asthana and Kiefer [47], and de Vreede et al. [48]. Figure 2 presents a Voigt function for different values of Cauchy and Gauss integral breadths.

Integral breadth of the Voigt function is expressed through its constituent integral breadths [49]

$$\beta =\beta_{G}\frac{\exp(-k^{2})}{\text{erfc}(k)}.$$ (11)

Here, erfc denotes the complementary error function.

Because convolution of two Voigt functions is also a Voigt function, integral breadths are easily separable conforming to Eqs. (7) and (8).

#### 2.1.2 Separation of Size and Strain Broadening

After removing the instrumental broadening from the observed line profile, it is possible to analyze the pure-specimen (physically) broadened line profile, to consider the origins and amount of broadening.

In 1918 Scherrer [50] recognized that breaking the crystal into domains smaller than ~1000 Å causes diffraction-line broadening

$${\langle D\rangle }_{v}=\frac{K\lambda }{\beta (2\theta )\cos\theta }.$$ (12)

The constant *K* depends on the crystallite shape [51,52,53,54,55,56], but generally is close to unity. The main characteristic of size broadening is that it is independent of the reflection order, that is, independent of diffraction angle.

Most of the work on x-ray diffraction line broadening was done on metals and alloys. It is widely accepted that plastic deformation in metals produces dislocation arrays, which divide crystallites into much smaller incoherently scattering domains. These dislocations produce strains within the domains, causing strain broadening. It was elaborated in Sec. 1.2 that any lattice imperfection (vacancies, interstitials, and substitutions) would broaden the diffraction peaks. These effects would be interpreted in the frame of this theory as a strain broadening, too. Stokes and Wilson [57] defined “apparent strain” as

η=β(2θ)cotθ. (13)

Strain broadening is angle dependent. Therefore, the angle dependence of the line broadening gives a possibility to distinguish between contributions of size and strain. However, when we speak of size and strain broadening, they may include other contributions. For instance, stacking faults and twins will contribute to broadening similar to size effects.

##### Warren-Averbach Method

**Fig. 3:**
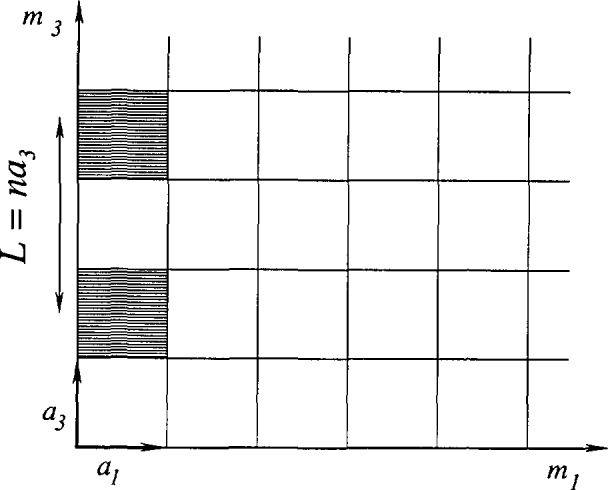
Representation of the crystal in terms of columns of cells along the *a*_3_ direction [59],

This method was developed originally for plastically deformed metals, but since its introduction [58, 22] it found successful application to many other materials. The method is extensively described in Warren’s publications [59, 60]. Each domain is represented by columns of cells along the 03 direction [61] (see Fig. 3). The crystal has orthorhombic axes with the direction 03 normal to the diffracting planes (00*l*). The experimentally observable diffraction power may be ex-pressed as a Fourier series

$$I(2\theta )=\frac{c}{\sin^{2}\theta }\sum_{n=-\infty }^{\infty }A_{n}\exp(2\pi ina_{3}s).$$ (14)

Here, experimentally measurable coefficients *A_n_* are

$$A_{n}=\frac{N_{n}}{{\langle D\rangle }_{s}}\langle \exp(2\pi ilZ_{n})\rangle .$$ (15)

The *A_n_* coefficients are the product of two terms. The first term depends only on the column length (size coefficient); the second depends only on distortion in domains (distortion coefficient):

$$A_{n}=A_{n}^{S}A_{n}^{D}.$$ (16)

$$A_{n}^{S}=\frac{N_{n}}{{\langle D\rangle }_{s}};A_{n}^{D}=\langle \exp(2\pi ilZ_{n})\rangle .$$ (17)

It is more convenient to express the distortion coefficient in terms of the strain component. If *L* = *na*_3_ is the undistorted distance between a pair of cells along direction *a*_3_, and distortion changes distance by *ΔL* = *a*_3_*Z_n_*, the component of strain in the as direction (orthogonal to reflecting planes) averaged over distance *L* can be defined as (*L*) = *Δ*(*L*)/*L*. Because *a*_3_*/l = d*, interplanar spacing, the distortion coefficient can be rewritten

$$A^{D}(L)=\langle \exp(2\pi iL\epsilon (L)/d)\rangle .$$ (18)

To obtain the strain component, it is necessary to approximate the exponential term. For not too large *L*

$$\begin{array}{c} \langle \exp(2\pi iL\epsilon (L)/d\rangle \\ \approx \exp(-2\pi^{2}L^{2}\langle \epsilon^{2}(L)\rangle /d^{2}). \end{array}$$ (19)

This relationship is exact if the distributions of *ϵ*(*L*) for all *L* values follow the Gauss function and is generally true as far as terms in *ϵ*^3^(*L*) because these distributions are usually sufficiently symmetrical [57]. Now, Eq. (16) can be approximated as

$$\ln A(L)=\ln A^{S}(L)-(2\pi^{2}\langle \epsilon^{2}(L)\rangle L^{2}/d^{2}).$$ (20)

Warren and Averbach [22] derived this relationship in a similar way. It separates size and strain contributions to the broadening, and allows for their simultaneous evaluation.

If the size coefficients are obtained by applications of Eq. (20), it is possible to evaluate the average surface-weighted domain size and the surface-weighted column-length distribution function [59]:

$${\left(\frac{dA^{S}(L)}{dL}\right)}_{L\to 0}=\frac{1}{{\langle D\rangle }_{s}};$$ (21)

$$P_{s}(L)\propto \frac{d^{2}A^{S}(L)}{dL^{2}}.$$ (22)

**Fig. 4:**
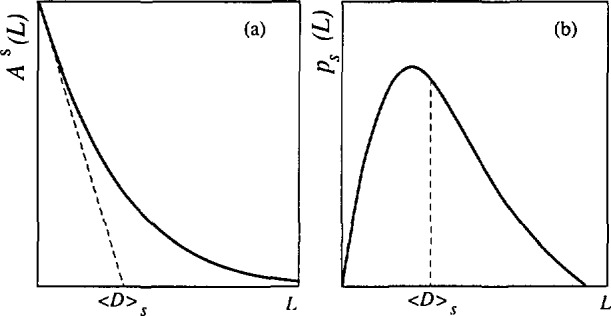
Surface-weighted domain size is determined: (a) by the intercept of the initial slope on the L-axis; (b) as a mean value of the distribution function.

Figure 4 shows how 〈*D*〉_s_, can be obtained from both the size coefficients *A*^S^(*L*) and the column-length distribution function.

##### Multiple-Line Integral-Breadth Methods

To separate size and strain broadening by using integral breadths, it is necessary to define the functional form for each effect. In the beginning, size and strain contributions were described by Cauchy or Gauss functions. Using Eqs. (12) and (13) on the *s* scale, and additive relations for the integral breadths following Eqs. (7) and (8)

$$\beta =\frac{1}{{\langle D\rangle }_{v}}+2es\;(\text{Cauchy-Cauchy}),$$ (23)

$$\beta =\frac{1}{{\langle D\rangle }_{v}}+\frac{4e^{2}s^{2}}{\beta }\;(\text{Cauchy-Gauss}),$$ (24)

$$\beta^{2}=\frac{1}{{\langle D\rangle }_{v}^{2}}+4e^{2}s^{2}\;(\text{Cauchy-Gauss}).$$ (25)

Here, *e = η*/4 ≈ *Δd*/*d* is the upper limit for a strain. Equation (24) uses the Haider and Wagner [62] parabolic approximation for the integral breadth of the Voigt function expressed by Eq. (11):

$$\frac{\beta_{C}}{\beta }=1-{\left(\frac{\beta_{G}}{\beta }\right)}^{2}.$$ (26)

Experience shows, however, that neither Cauchy nor Gauss functions can model satisfactorily size or strain broadening in a general case. Langford [26] introduced the so-called multiple-line Voigt-function analysis. Both size-broadened and strain-broadened profiles are assumed to be Voigt functions. Using Eqs. (12) and (13), it follows symbolically for Cauchy and Gauss parts that

$$\beta_{C}=\beta_{\text{SC}}+\beta_{\text{DC}}s;$$ (27)

$$\beta_{G}^{2}=\beta_{\text{SG}}^{2}+\beta_{\text{DG}}^{2}s^{2}.$$ (28)

This approach disagrees with the Warren-Averbach analysis, that is, the two methods give different results (see Sec. 4.4) [63, 64].

##### Single-Line Methods

There are cases where only the first order of reflection is available or higher-order reflections are severely suppressed (extremely deformed materials, multiphase composites, catalysts, and oriented thin films). Many methods exist to separate size and strain broadening from only one diffraction peak. However, it was stated in Sec. 2.1.2 that the different size and strain broadening angle dependence is a basis for their separation; hence, using only one diffraction line introduces a contradiction. Consequently, single-line methods should be used only when no other option exists. The single-line methods can be di-vided in two main parts: Fourier-space and real-space methods. Fourier-space methods are based on the Warren-Averbach separation of size and strain broadening following Eq. (20). The functional form of 〈*E*^2^(*L*)〉 is assumed either to be constant [65,66,67,68], or assumed to depend on *L* as 〈*ϵ*^2^(*L*)〉*=c/L* [69,70,71,72]. Then, Eq. (20) can be fitted to few points *of A*(*L*) for the small averaging distance *L*, to obtain size and strain parameters. All Fourier-space methods have the serious problem that the Fourier coefficients., *A*(*L*) are usually uncertain for small *L*, because of the so-called “hook” effect [60] (see Sec. 4.2). Zocchi [73] suggested that fitting the straight line through the first derivatives of the Fourier coefficients, instead of through the coefficients themselves, would solve the “hook”-effect problem.

All real-space methods [74,75,76] are based on the assumption that the Cauchy function deter-mines size and that the Gauss function gives strain. The most widely used method of de Keijser et al. [76] gives size and strain parameters from Cauchy and Gauss parts of the Voigt function, respectively:

$${\langle D\rangle }_{v}=\frac{1}{\beta_{C}};$$ (29)

$$e=\frac{\beta_{G}}{2s}.$$ (30)

### 2.2 Diffraction-Line-Broadening Analysis of Superconductors

In this field, very few studies exist. Williams et al. [77] reported isotropic strains in YBa_2_Cu_3_O_7−_*_δ_* powder by the simultaneous Rietveld refinement of pulsed-neutron and x-ray diffraction data. Using a GSAS Rietveld refinement program [78], both size and strain broadening were modeled with the Gauss functions for the neutron-diffraction data [79, 80], and with the Cauchy functions for the x-ray diffraction data (modified method of Thompson, Cox, and Hastings [81]). Interestingly, both the neutron and x-ray data gave identical values for the isotropic strain (0.23%) and no size broadening. Singh et al. [82] studied internal strains in YBa_2_Cu_3_O_7−_*_δ_* extruded wires by pulsed-neutron diffraction. They separated size and strain parameters by means of Eq. (25) (Gauss-Gauss approximation). Size broadening was found to be negligible, but (isotropic) microstrains range from 0.05% for the coarse-grained material to 0.3% for the fine-grained samples. Eatough, Ginley, and Morosin [83] studied Tl_2_Ba_2_Ca_2_Cu_3_O_10_ (Tl-2223) and Tl_2_Ba_2_CaCu_2_O_8_ (Tl-2212) superconducting thin films by x-ray diffraction. Using the Gauss-Gauss approximafion, they found strains of 0.14–0.18% in both phases, and domain sizes of 1200-1400 Å for Tl-2212, but 500 Å for Tl-2223.

We are aware of only two more unpublished studies [84, 85] involving size-strain analysis in high-*T*_c_ superconductors. The probable reason is that any analysis is very difficult because of weak line broadening and overlapping reflections. This precludes application of reliable analysis, such as the Stokes deconvolution method with the Warren-Averbach analysis of the broadening. Instead, simple integral-breadth methods are used, which gives generally different results for each approach. Moreover, for x-ray diffraction broadening, application of the Gauss-Gauss approximation does not have any theoretical merit, although reasonable values, especially of domain sizes, may be obtained [86]. We showed [87,88,89] that reliable diffraction-line-broadening analysis of superconductors can be accomplished and valuable information about anisotropic strains and incoherently diffracting domain sizes obtained.

## 3. Experiment

### 3.1 Materials

The materials used for this study were tungsten and silver commercially available powders with nominal grain sizes 4-12 μm, La_2-_*_x_*Sr*_x_*CuO_4_(*x* = 0, 0.06, 0.15, 0.24) powders, La_1.85_M_0.15_CuO_4_(M = Ca, Ba) powders, Bi_2_Sr_2_CaCu_2_O_8_ (Bi-2212) sinter, (BiPb)_2_Sr_2_Ca_2_Cu_3_O_10_(Bi,Pb-2223) sinter, and (BiPb)_2_(SrMg)_2_(BaCa)_2_Cu_3_O_10_ (Bi,Pb,Mg,Ba-2223) sinter.

Powders with nominal compositions La_2-_*_x_*Sr*_x_*CuO_4_(*x* = 0, 0.06, 0.15, 0.24) and La_1.85_M_0.15_CuO_4_(M = Ca, Ba) were prepared at the National Institute of Standards and Technology, Boulder, Colorado, by A. Roshko, using a freeze-drying acetate process [90]. Acetates of the various cations were assayed by mass by calcining to the corresponding oxide or carbonate. The appropriate masses of the acetates for the desired compositions were dissolved in deionized water. The acetate solutions were then sprayed through a fine nozzle into liquid nitrogen to preserve the homogeneous cation distributions. Frozen particles were transferred to crystallization dishes and dried in a commercial freeze dryer, to a final temperature of 100 °C. After drying, the powders, except the La_1.85_M_0.15_CuO_4_, were calcined in alumina (99.8%) crucibles at 675 °C for 1 h in a box furnace with the door slightly open to increase ventilation. Because BaCO_3_ is difficult to decompose, the La_1.85_M_0.15_CuO_4_ was calcined under a vacuum of 2 Pa at 800 °C for 4 h, then cooled slowly in flowing oxygen (2 °C/min). The calcined powders were oxidized in platinum-lined alumina boats in a tube furnace with flowing oxygen at 700 °C. After 3 h at 700 °C the powders were pushed to a cold end of the furnace tube where they cooled quickly (20 °C/s) while still in flowing oxygen.

The cylindrical specimens (23 mm in diameter and 9 mm thick) of Bi-2212, Bi,Pb-2223, and Bi,Pb,Mg,Ba-2223 were prepared at the National Research Institute for Metals, Tsukuba, Japan, by K. Togano [91], Starting oxides and carbonates were: Pb_3_O_0_, Bi_2_O_3_, CuO, SrCO_3_, MgCO_3_, and BaCO_3_. They were calcinated in air at 800 °C for 12 h. Powders were then pressed and sintered in 8% oxygen-92% argon mixture at 835 °C for 83 h. Specimens were furnace cooled to 750 °C, held for 3 h in flowing oxygen, and then furnace cooled in oxygen to room temperature.

#### 3.1.1 Preparation of Specimens for X-ray Diffraction

The bulk specimens were surface polished, if necessary, and mounted in specimen holders. Coarse-grained powders of La_2-_*_x_*Sr*_x_*CuO_4_ (*x* = 0, 0.06, 0.15, 0.24) and La_1.85_M_0.15_CuO_4_ (M = Ca, Ba) were ground with a mortar and pestle in toluene and passed through a 635-mesh sieve (20-μm nominal opening size). Silver and tungsten powders were dry ground with a mortar and pestle. All powders were mixed with about 30% silicone grease and loaded into rectangular cavities or slurried with amyl acetate on a zero-background quartz substrate.

### 3.2 Measurements

**Fig. 5:**
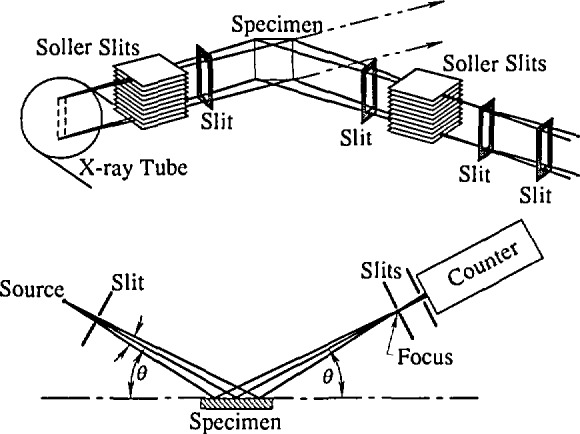
Optical arrangement of an x-ray diffractometer. Adapted from Klug and Alexander [24].

X-ray-diffraction data were collected using a standard two-circle powder goniometer in Bragg-Brentano parafocusing geometry [92, 93] (see Fig. 5). A flat sample is irradiated at some angle incident to its surface, and diffraction occurs only from crystallographic planes parallel to the specimen surface. The goniometer had a vertical *θ–2θ* axis and 22 cm radius. *CnKα* radiation, excited at 45 kV and 40 mA, was collimated with Seller slits [94] and a 2 mm divergence slit. Soller slits in the diffracted beam, 0.2 mm receiving slit and Ge solid-state detector were used in a step-scanning mode (0.01°/10 s for a standard specimen, 0.02°–0.05°/30-80 s for other specimens, depending on the amount of broadening).

### 3.3 Data Analysis

The diffractometer was controlled by a computer, and all measurements were stored on hard disc. Data were transferred to a personal computer for processing.

We used computer programs for most calculations. X-ray diffraction patterns were fitted with the program SHADOW [95]. This program allows a choice of the fitting function and gives refined positions of the peak maximums, intensities, and function-dependent parameters. It also has the ability to convolute the predefined instrumental profile with the specimen function to match the observed pattern. Choice of the specimen function includes Gauss and Cauchy functions. We added the ability to model the specimen broadening with an exact Voigt function and implemented SHADOW on a personal computer. In the fitting procedure, for every peak in the pattern, the program first generates the instrumental profile at the required diffraction angle. The instrumental profile is determined from prior measurements on a well-annealed standard specimen (see Sec. 5.1). Then it assumes parameters of the specimen profile. For an exact Voigt function, parameters are peak position, peak intensity, and Cauchy and Gauss integral breadths of the Voigt function. By convoluting the instrumental profile with the specimen profile, and adding a background, the calculated pattern is obtained [Eq. (2)]. Parameters of the specimen profile are varied until the weighted least-squares error of calculated and observed patterns Eq. (59), reaches a minimum. This process avoids the unstable Stokes deconvolution method. It is possible that the refinement algorithm is being trapped in a false minimum [96], but it can be corrected by constraining some parameters. Refined parameters of the pure-specimen profile are input for the size-strain analysis of the broadening. A program for this analysis was written in Fortran.

Lattice parameters of powder specimens were calculated by the program NBS*LSQ85, based on the method of Appleman and Evans [97]. A program in Fortran was written to apply corrections to observed peak maximums by using NIST standard reference material 660 LaB_6_ as an external standard. Lattice parameters of bulk specimens were determined by the Fortran program, which uses a modified Cohen’s method [98,99,100] to correct for systematic diffractometer errors. Lattice parameters were also calculated by the Rietveld refinement programs GSAS [78] and DBW3.2S [101, 102].

## 4. Methodology

When instrumental and specimen contributions to the observed line profile must be modeled separately, adopting a specimen function is a critical step. Yau and Howard [103,104] used Cauchy, and Enzo et al. [105] pseudo-Voigt functions. Benedetti, Fagherazzi, Enzo, and Battagliarin [106] showed that modeling the specimen function with the pseudo-Voigt function gives results comparable to those of the Stokes deconvolution method when combined with the Warren-Averbach analysis of Fourier coefficients. De Keijser, Mittemeijer, and Rozendaal [107] analytically derived domain sizes and root-mean-square strains for small averaging distance *L* in the case of the Voigt and related functions.

The aim here is to study more thoroughly the consequences of assumed Voigt specimen function on the size-strain analysis of the Fourier coefficients of the broadened peaks. It is shown that some experimentally observed interrelations between derived parameters (particularly volume-weighted and surface-weighted domain sizes) and their behavior (the “hook” effect and dependence of mean-square strains on the averaging distance) can be explained by this simple assumption. Moreover, the discrepancy between the integral-breadth methods and Warren-Averbach analysis results from different approximations for the strain broadening and the background experimental errors.

### 4.1 Separation of Size and Strain Broadenings

The normalized Fourier transform of a Voigt function is easily computable [46]:

$$A_{n}=\exp\left[-2n\frac{\beta_{C}(2\theta )}{\sigma (2\theta )}-\pi n^{2}\frac{\beta_{B}^{2}(2\theta )}{\sigma^{2}(2\theta )}\right].$$ (31)

It is convenient to express Fourier coefficients in terms of distance *L*, by immediately making the approximation *Δ*(*2θ*) *= λΔs*/cos*θ*_0_:

$$A(L)=\exp[-2L\beta_{C}-\pi L^{2}\beta_{G}^{2}].$$ (32)

Equation (32) is a good approximation even for large specimen broadening. Even for a profile span of *Δ*(2*θ*) = 80°, the error made by replacing this interval by an adequate *Δ*(sin *θ*) range is 2%. However, strictly speaking, the profile will be asymmetrical in reciprocal space, and Fourier-interval limits will not correspond to the 2*θ*_1_ and 2*θ*_2_ peak-cutoff values in real space. It is important to keep Fourier interval limits identical for all multiple-order reflections; otherwise serious errors in the subsequent analysis will occur [108]. If higher accuracy for a considerable broadening is desired, profile fitting can be accomplished In terms of the reciprocal-space variable *s*, instead of in a real 2*θ* space.

Assuming that only the Cauchy function determines domain size [*A*^S^(*L*) = exp(−*L*/〈*D*)〉_s_)] and only the Gauss function gives root-mean-square strain (RMSS) [*A*^D^(*L*) = exp(−2*π*^2^*L*^2^〈*ϵ*^2^〉/*d*^2^)], Eq. (32) leads to the Warren-Averbach [Eq. (20)] for the separation of size and strain contribution [62]. Experience shows that Cauchy and Gauss functions can not satisfactory model specimen broadening. Balzar and Ledbetter [64] postulate that the specimen function includes contributions of size and strain effects, both approximated with the Voigt functions. Because the convolution of two Voigt functions is also a Voigt function, Cauchy and Gauss integral breadths of the specimen profile are easily separable:

$$\beta_{C}=\beta_{\text{SC}}+\beta_{\text{DC}};$$ (33)

$$\beta_{G}^{2}=\beta_{\text{SG}}^{2}+\beta_{\text{DG}}^{2}.$$ (34)

Langford [26] separated the contributions from size and strain broadening in a similar way. (See Eqs. (27) and (28).) Note, however, that Eqs. (33) and (34) do not define size and strain angular order-dependence.

Because Fourier coefficients are a product of a size and a distortion coefficient, from Eqs. (32), (33), and (34), we can obtain the separation of size and strain contributions to the pure specimen broadening:

$$A^{S}(L)=\exp(-2L\beta_{\text{SC}}-\pi L^{2}\beta_{\text{SG}}^{2});$$ (35)

$$A^{D}(L)=\exp(-2L\beta_{\text{DC}}-\pi L^{2}\beta_{\text{DG}}^{2}).$$ (36)

Wang, Lee, and Lee [109] modeled the distortion coefficient, and Selivanov and Smislov [110] modeled the size coefficient in the same way.

To obtain size and distortion coefficients, at least two reflections from the same crystallographic-plane family must be available.

### 4.2 Size Coefficient

Surface-weighted domain size is calculated from the size coefficients following Eq. (21). From Eq. (35) we obtain

$${\langle D\rangle }_{s}=\frac{1}{2\beta_{\text{SC}}}.$$ (37)

Therefore, surface-weighted domain size depends only on the Cauchy part of the size-integral breadth.

The second derivative of the size coefficients is proportional to the surface-weighted column-length distribution function, Eq. (22). The volume-weighted column-length distribution function follows similarly [111]:

$$p_{v}(L)\propto L\frac{d^{2}A^{A}(L)}{dL^{2}.}$$ (38)

By differentiating Eq. (35) twice, we obtain

$$\frac{d^{2}A^{S}(L)}{dL^{2}}=[{\left(2\pi L\beta_{\text{SG}}^{2}+2\beta_{\text{SC}}\right)}^{2}-2\pi \beta_{\text{SG}}^{2}A^{S}(L).$$ (39)

Because the column-length distribution function should always be positive [59], the Cauchy part must dominate. Inspection of Eq. (39) shows that for small *L* we must require

$$\beta_{\text{SC}}\geq \sqrt{\frac{\pi }{2}}\beta_{\text{SG}}.$$ (40)

**Fig. 6:**
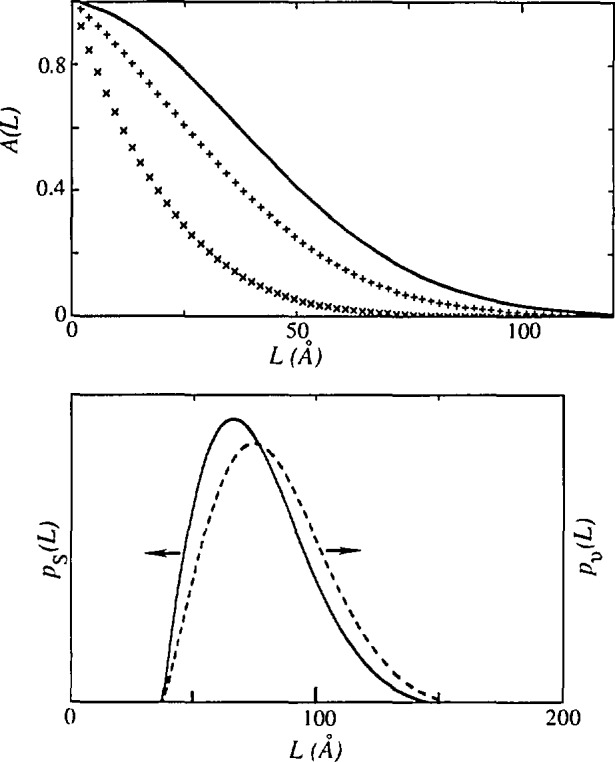
(upper) The “hook” effect of the size coefficients *A*^S^ (full line) at small *L*; (lower) it causes negative values (set to zero) of the column-length distribution functions.

Otherwise, the “hook” effect will occur in the plot of size coefficients *A*^S^(*L*) versus *L*, that is, the plot will be concave downward for small *L* (Fig. 6). The “hook” effect is usually attributed to experimental errors connected with the truncation of the line profiles, and consequently overestimation of background [59]. This is a widely encountered problem in the Fourier analysis of line broadening. It results in overestimation of effective domain sizes and underestimation of the RMSS [36]. Some authors [106] claim that the preset specimen-broadening function eliminates the “hook” effect. However, Eq. (39) shows that, effectively, too high background causes underestimation of the Cauchy content of the Voigt function, because the long tails are truncated prematurely. It has to be mentioned that Wilkens [112] proposed tilted small-angle boundaries to be the source of the “hook” effect. Liu and Wang [113] defined a minimum particle size present in a specimen, depending on the size of the “hook” effect. Figure 6 shows that negative values of the column-length distribution functions (set to zero), do not affect the shape, but shift the entire distribution toward larger *L* values.

Note also that the surface-weighted column-length distribution function *p*_s_(*L*) will usually have a maximum at *L* = 0; but for the particular ratio of integral breadths, determined by Eq. (40), it can be zero. The volume-weighted column-length distribution function *p*_v_(*L*) will always have a maximum for *L* ≠ 0.

If the column-length distribution functions are known, it is possible to evaluate mean values of respective distributions:

$${\langle D\rangle }_{s,v}=\frac{\int_{0}^{\infty }Lp_{s,v}(L)dL}{\int_{0}^{\infty }p_{s,v}(L)dL}.$$ (41)

Integrals of this type can be evaluated analytically [114]:

$$\begin{array}{c} \int_{0}^{\infty }x^{m}\exp(-bx^{2}-cx)dx=\frac{{\left(-1\right)}^{m}}{2}\sqrt{\frac{\pi }{b}}\frac{\partial^{m}}{\partial c^{m}}\\ \left[\exp(\frac{c^{2}}{4b}erfc\left(\frac{c}{2\sqrt{b}}\right)\right]. \end{array}$$ (42)

Surface-weighted domain size 〈D〉_s_ must be equal to the value obtained from Eq. (37). The volume-weighted domain size follows:

$${\langle D\rangle }_{v}=\frac{\exp{\left(k\right)}^{2}}{\beta_{\text{SG}}}\text{erfc}(k)=\frac{1}{\beta^{S}}.$$ (43)

Using Eqs. (37) and (43), we can evaluate the ratio of domain sizes:

$$\frac{{\langle D\rangle }_{v}}{{\langle D\rangle }_{s}}=2\sqrt{\pi }k\exp(k^{2})\text{erfc}(k).$$ (44)

Theoretically, *k* can change from zero to infinity. However, the minimum value of *k* is determined by Eq. (40):

$$1/\sqrt{2}\leq k<\infty .$$ (45)

**Fig. 7:**
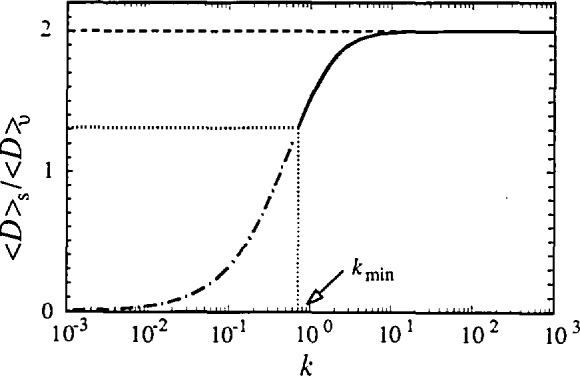
The ratio of volume-weighted and surface-weighted domain sizes as a function of the characteristic ratio of Cauchy and Gauss integral breadths *k*.

Hence, the ratio of domain sizes can change in a limited range (see also Fig. 7):

$$1.31\approx \sqrt{2\pi e}\text{erfc}\left(\frac{1}{\sqrt{2}}\right)\leq \frac{{\langle D\rangle }_{v}}{{\langle D\rangle }_{s}}<2.$$ (46)

It may be noted that most experiments give the ratio 〈*D*〉_v_*/*〈*D*〉_s_ in this range (see for instance re-view by Klug and Alexander [24]). When *k* goes to infinity the size broadening is given only by the Cauchy component and 〈*D*〉_v_ = 2〈*D*〉_s_. This is a case of pure Cauchy size broadening, described by Haider and Wagner [62] and de Keijser, Mittemeijer and Rozendaal [107]. It is possible to imagine a more complicated column-length distribution function [27] than Eq. (39), which would allow even larger differences between surface-weighted and volume-weighted domain sizes. However, we are not aware of any study reporting a difference larger than 100%.

### 4.3 Distortion Coefficient

In Sec. 2.1.2 it was shown that the distortion co-efficient can be approximated by the exponential

$$A^{D}(L)=\exp(-2\pi^{2}s^{2}L^{2}\langle \epsilon^{2}(L)\rangle ).$$ (47)

Comparing with Eq. (36), we can write

$$\langle \epsilon^{2}(L)\rangle =\frac{1}{s^{2}}\left(\frac{\beta_{\text{DG}}^{2}}{2\pi }+\frac{\beta_{\text{DC}}}{\pi^{2}}\frac{1}{L}\right).$$ (48)

Therefore, mean-square strains (MSS) decrease linearly with averaging distance *L*. This behavior is usually observed in the Warren-Averbach analysis. Rothman and Cohen [115] showed that such behavior would be expected of strains around dislocations. Adler and Houska [116], Houska and Smith [117], and Rao and Houska [118] demonstrated for a number of materials that MSS can be represented by a sum of two terms, given by Cauchy and Gauss strain-broadened profiles.

However, for *β*_DC_ = 0, the MSS are independent of *L*:

$${\langle \epsilon^{2}\rangle }^{1/2}=\frac{\beta_{\text{DG}}(2\theta )}{2\sqrt{2\pi }}\cot\theta =\frac{2}{\sqrt{2\pi }}e,$$ (49)

where the upper limit of strain *e* is defined as *η*/4 (see Sec. 2.1.2). This is a limiting case of pure-Gauss strain broadening, described by de Keijser, Mittemeijer and Rozendaal [107].

### 4.4 Discussion

To calculate domain sizes and strain, it is necessary to define size and distortion integral-breadths angular order-dependence. From Eq. (43) It follows that the domain size 〈*D*〉_v_ is always independent of the order of reflection:

$$\beta_{\text{SC}}=\text{const}.;\;\beta_{\text{SG}}=\text{const}.$$ (50)

However, from Eq. (48) we find

$$\frac{\beta_{\text{DC}}}{s^{2}}=\text{const}.;\;\frac{\beta_{\text{DG}}}{s}=\text{const}.$$ (51)

An important consequence is that “apparent strain” *η* will be independent of angle of reflection only in the case of pure-Gauss strain broadening because *β*_DC_ and *β*_DG_ depend differently on the diffraction angle. If we compare Eq. (51) with the multiple-line Voigt-function analysis [26], given by Eqs. (27) and (28), it is evident that they disagree. Therefore, recombination of constituent integral breadths *β*_DC_ and *β*_DG_ to Voigt strain-broadened integral breadth *β*^D^ and the subsequent application of Eq. (49) to calculate strain [26, 119] will concur with the Fourier methods if strain broadening is entirely Gaussian and for the asymptotic value of MSS (〈*ϵ*^2^(∞)〉). However, neither volume-weighted domain sizes 〈*D*〉_v_ will agree, although they are de-fined identically in both approaches, because size-broadened and strain-broadened integral breadths are dependent variables.

If at least two orders of reflection (*l* and *l* +1) of the same plane (*hkl*) are available, using Eqs. (50) and (51) we can solve Eqs. (33) and (34):

$$\beta_{\text{SC}}=\frac{s^{2}\left(l+1\right)\beta_{C}\left(l\right)-s^{2}\left(l\right)\beta_{C}\left(l+1\right)}{s^{2}\left(l+1\right)-s^{2}\left(l\right)}$$ (52)

$$\beta_{\text{SG}}^{2}=\frac{s^{2}(l+1)\beta_{G}^{2}(l)-s^{2}(l)\beta_{G}^{2}(l+1)}{s^{2}(l+1)-s^{2}(l)}$$ (53)

$$\beta_{\text{DC}}=\frac{s^{2}(l)}{s^{2}(l+1)-s^{2}(l)}[\beta_{C}(l+1)-\beta_{C}(l)]$$ (54)

$$\beta_{\text{DG}}^{2}=\frac{s^{2}\left(l\right)}{s^{2}\left(l+1\right)-s^{2}\left(l\right)}\left[\beta_{\text{G}}^{2}\left(l+1\right)-\beta_{\text{G}}^{2}\left(l\right)\right].$$ (55)

If we substitute these expressions into Eqs. (35) and (36), we see that this approach leads exactly to the Warren-Averbach Eq. (20) for two orders of reflections. This is expected because the distortion coefficient is approximated with the exponential (47)]. Delhez, de Keijser, and Mittemeijer [23] argued that, instead of Eq. (20), the following relation would be more accurate:

$$A(L)=A^{s}(L)(1-2\pi^{2}\langle \epsilon^{2}(L)\rangle L^{2}/d^{2}).$$ (56)

These two approximations differ with fourth-order terms in the power-series expansion. In terms of this approach, Eq. (48) has to be rewritten:

$$\langle \epsilon^{2}(L)\rangle =\frac{1-\exp(-2L\beta_{\text{DC}}-\pi L^{2}\beta_{\text{DG}}^{2})}{2\pi^{2}s^{2}L^{2}}.$$ (57)

**Fig. 8:**
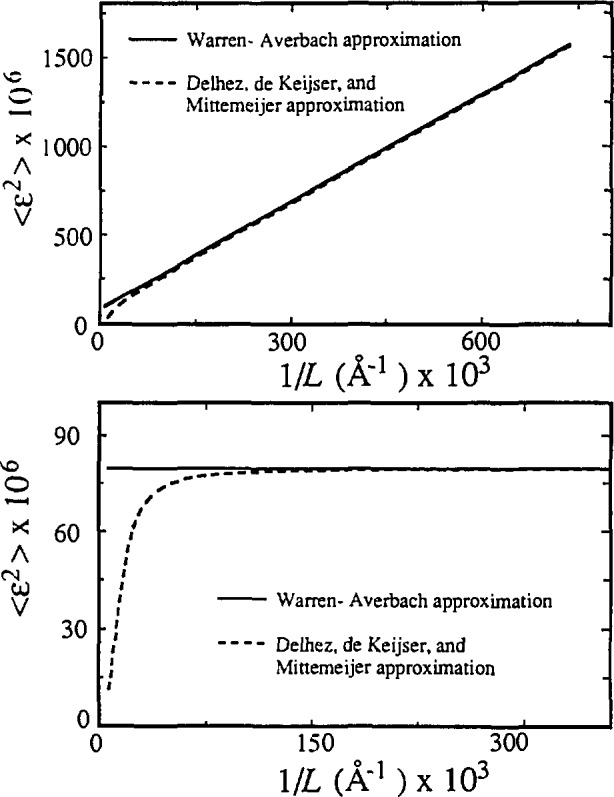
Mean-square strains 〈*ϵ*^2^(*L*)〉 for two approximations of the distortion coefficient: (upper) Voigt strain broadening; (lower) pure-Gauss strain broadening.

This means that even if the strain-broadened pro-file is given entirely by the Gauss function, the MSS depend on distance *L* (see Fig. 8). In this approximation no simple relation for the distortion integral-breadths angular order-dependence exists. For not so large *L*, however, Eq. (51) holds, and approximations from Eqs. (20) and (56) do not differ much (see Fig. 8).

Generally, it was shown that in the size-broadened profile the Cauchy part must dominate. No similar requirement for the strain-broadened profile exists. However, experience favors the assumption that it has to be more of Gauss-type. The Warren-Averbach approach is exact if strain broadening is purely Gaussian, so Eqs. (20) and (48) are better approximations as strain profile is closer to the Gauss function. In any case, both approaches, given by Eqs. (20) and (56), are good up to the third power in strain, and the Warren-Averbach relation [Eq. (20)] does not assume that MSS are independent of distance *L* [27]; it also represents a harmonic approximation.

If at least two orders of reflection from the same plane (*hkl*) are available, we can use Eqs. (52), (53), (54), and (55) to calculate size-related and strain-related integral breadths. Subsequent application of Eqs. (37), (43), and (48) gives directly domain sizes 〈*D*〉, and 〈D〉_v_, and mean-square strains (*ϵ*^2^(*L*)). This approach is more straightforward and much simpler than the original Warren-Averbach analysis. Great care should be given to the possible systematic errors. The easiest way to observe the “hook” effect is to plot column-length distribution functions as a function of averaging distance *L*. If they show negative values for small *L* (see Fig. 6), all derived parameters will be in error. This is because (i) only the positive values of the column-length distribution functions are numerically integrated or (ii) the intercept on the *L*-axis of the linear portion of the A^S^ vs *L* curve is taken (see Fig. 4), always larger values of domain sizes will be obtained than by the application of Eqs. (37) and (43). Therefore, we conclude that the discrepancy between integral-breadth and Fourier methods is always present by the appearance of the “hook” effect in the A^S^ vs *L* curve. An analogous discrepancy exists between integral-breadth and variance methods [119]. In such cases, correction methods for truncation can be applied [35, 119, 120], but the best procedure is to repeat the pattern fitting with the correct background.

In the Fourier analysis it is usually observed that the mean-square strains diverge as the averaging distance *L* approaches zero. This also follows from Eqs. (48) and (57). However, because the MSS dependence on distance *L* is not defined in Warren-Averbach analysis, it was suggested [27, 107, 121] that local strain can be obtained by taking the second derivative of the distortion coefficient, or by a Taylor-series expansion of local strain. Therefore, we obtain from Eq. (36):

$$\langle \epsilon^{2}(0)\rangle =\frac{1}{s^{2}}\left(\frac{\beta_{\text{DG}}^{2}}{2\pi }-\frac{\beta_{\text{DC}}^{2}}{\pi^{2}}\right).$$ (58)

It is evident that this relation is wrong. It holds only for a special case of pure-Gauss strain-broadened profile, when the MSS are equal for any *L*. Otherwise, if the Cauchy function contributes to strain broadening, all derivatives of strain in *L* = 0 are infinite, and local strain can not be defined. If the main origin of strains is dislocations [115], strains are defined after some distance from the dislocation (cutoff radius) to be finite. Averaging strains over a region smaller than the Burgers vector is probably not justified. For instance, Eq. (48) gives, even for a small averaging distance, *L* = 1 Å, and considerable strain broadening (*β*_DG_(2*θ*) = *β*_DC_(2*θ*) = 10°), root-mean-square strain 〈*ϵ*^2^*L* = 1 Å)〉^1/2^ ≈ 0.2, that is, about the elastic limit.

### 4.5 Random Errors of Derived Parameters

Errors in size and strain analysis of broadened peaks are relatively difficult to evaluate. Following Langford [26], sources of the systematic errors include choice of standard specimen, background, and type of analytical function used to describe the line profiles. The first two errors should be minimized in the experimental procedure. Errors caused by inadequate choice of specimen function would systematically affect all derived results, but they can not be evaluated. Random errors caused by counting statistics have been computed by Wilson [122, 123, 124] and applied to the Stokes deconvolution method by Delhez, de Keijser, and Mittemeijer [23], as well as by Langford [26] and de Keijser et al. [76] using single-line Voigt-function analysis. Nevertheless, the approximate error magnitude can be calculated from estimated standard deviations (e.s.d.) of the parameters refined in the fitting procedure. In the program SHADOW, the weighted least-squares error is minimized:

$$R_{\text{wp}}=\frac{RES}{\sum_{i=1}^{m}w_{i}I_{i}^{2}(\text{obs})}.$$ (59)

Here

$$RES=\sum_{i=1}^{m}w_{i}{\left[I_{i}(\text{obs})-I_{i}(\text{cal})\right]}^{2}$$ (60)

and weights are the reciprocal variances of the observations:

$$w_{i}=1/I_{i}(\text{obs}).$$ (61)

Each line profile has four parameters varied independently: position, intensity, and Cauchy and Gauss integral breadths of the Voigt profile. In least-squares refinement, e.s.d.’s are computed as

$$\sqrt{b_{ii}\frac{RES}{m-m^{\prime }}}.$$ (62)

Here *b*_ii_ are diagonal elements of the inverse matrix of the equation coefficients, *m* is the number of observations, and *m*′ is the number of refined parameters. The main source of errors is integral breadths. Errors in peak position, peak intensity, and background are much smaller and can be neglected in this simple approach. For two independent variables, *β*_G_ and *β*_C_, covariance vanishes, and from Eqs. (37) and (38), for the two orders of re-flection, *l* and *l* +1, it follows that

$$\begin{array}{c} R^{2}({\langle D\rangle }_{s}=\\ \frac{s^{4}(l+1)\beta_{C}^{2}(l)R^{2}(\beta_{C}(l))+s^{4}(l)\beta_{C}^{2}(l+1)R^{2}(\beta_{C}(l+1))}{{\left[s^{2}(l+1)-\beta_{C}(l)-s^{2}(1)-\beta_{C}(l+1)\right]}^{2}}; \end{array}$$ (63)

$$\begin{array}{c} R^{2}\left({\langle \epsilon^{2}(L)\rangle }^{1/2}\right)=\\ \frac{\beta_{C}^{2}(l)R^{2}(\beta_{C}(l))+\beta_{C}^{2}(l+1)R^{2}(\beta_{C}(l+1))}{{\left\{2\left[\beta_{C}(l+1)-\beta_{C}(l)\right]+\pi L\left[\beta_{G}^{2}(l+1)-\beta_{G}^{2}(l)\right]\right\}}^{2}}\\ +\frac{\pi^{2}L^{2}\left[\beta_{G}^{4}(l)R^{2}(\beta_{G}(l))+\beta_{G}^{4}(l+1)R^{2}(\beta_{G}(l+1))\right]}{{\left\{2[\beta_{C}(l+1)-\beta_{C}(l)+\pi L\left[\beta_{G}^{2}(l+1)-\beta_{G}^{2}(l)\right]\right\}}^{2}}. \end{array}$$ (65)

Here *R*(*x*) are “relative standard deviations.” The error in 〈*D*〉_v_ would be complicated to evaluate, but because 1.31〈*D*〉_s_ ≤ 〈*D*〉_v_ < 2〈*D*〉_s_, Eq. (63) gives a good estimation for the error in 〈*D*〉_v_ as well. Alter-natively, to see how errors depend on the Fourier coefficients, errors can be estimated from the Warren-Averbach relationship [Eq. (20)] (86). From Eq. (32) it follows that

$$R^{2}(A(L))=4\pi^{2}L^{4}\beta_{G}^{4}(s)R^{2}(\beta_{G}(2\theta ))+4L^{2}\beta_{C}^{2}(s)R^{2}(\beta_{C}(2\theta )).$$ (66)

Errors in root-mean-square strains and domain sizes are

$$\begin{array}{c} R^{2}\left({\langle \epsilon^{2}(L)\rangle }^{1/2}\right)=\\ \frac{R^{2}(A(L,l))+R^{2}(A(L,l+1))}{4\ln^{2}\frac{A(L,l)}{A(L,l+1)}}; \end{array}$$ (67)

$$\begin{array}{c} R^{2}\left({\langle D\rangle }_{s}\right)={\left(\frac{A^{S}(L)}{1-A^{S}(L)}\right)}^{2}\\ \frac{s^{4}(l+1)R^{2}(A(L,l))+s^{4}(l)R^{2}(A(L,l+1))}{{\left[s^{2}(l+1)-s^{2}(l)\right]}^{2}}. \end{array}$$ (68)

Here, 〈*D*〉_s_, is approximately defined with A^S^(*L*) = l − *L*/〈*D*〉_s_. Errors in Fourier coefficients increase with *L*, while factors in Eqs. (67) and (68) lower the errors for large *L*. In general, errors of domain sizes and strains are of the same order of magnitude as errors of integral breadths [86].

## 5. Application

### 5.1 Correction for Instrumental Broadening

Before specimen broadening is analyzed, instrumental broadening must be determined. This is accomplished by carefully measuring diffraction peaks of some well-annealed “defect-free” specimen. It is then assumed that its broadening may be attributed only to the instrument. The usual procedure is to anneal the specimen. However, in some instances that is not possible, because either the material undergoes an irreversible phase transition on annealing, or the number of defects can not be successfully decreased by annealing. Another possibility is to measure the whole diffraction pattern of the material showing the minimal line broadening, and then to synthesize the instrumental profile at the needed diffraction angle. This approach re-quires the modeling of the angle dependence of the instrumental (standard) parameters. Cagliotti, Paoletti, and Ricci [125] proposed the following function to describe the variation of the full width at the half maximum of profile with the diffraction angle:

$${\text{FWHM}}^{2}(2\theta )=U\tan^{2}\theta +V\tan\theta +W.$$ (69)

Although this function was derived for neutron diffraction, it was confirmed to work well also in x-ray diffraction case [126, 127]. A more appropriate function for the x-ray angle-dispersive powder diffractometer, based on theoretically predicted errors of some instrumental parameters [128] may be the following [129]:

$${\text{FWHM}}^{2}(2\theta )=W+V\sin^{2}2\theta +U\tan^{2}\theta +U^{\prime }\cot^{2}\theta .$$ (70)

This function may better model the increased axial divergency at low angles and correct for the specimen transparency [129]. However, contrary to the requirement on the specimen function, most important for the instrumental function is to correctly describe the angular variation of parameters, regardless of its theoretical foundation.

When specimen broadening is modeled with a Voigt function, the simplest way to correct for the instrumental broadening is by fitting the line profiles with the Voigt function, too. Cauchy and Gauss integral breadths of the specimen-broadened profile are then easily computable by Eqs. (7) and (8). However, because the instrumental broadening is asymmetric [24], modeling with the symmetric Voigt function can cause a fictitious error distribution, resulting in errors of strain up to 35% [76]. Another approach is to model the instrumental-broadening angle dependence by fitting the profile shapes of a standard specimen with some asymmetrical function; split-Pearson-VII [95] or pseudo-Voigt convoluted with the exponential function [105]. The instrumental function can then be synthesized at any desired angle of diffraction and convoluted with the assumed specimen function to match the observed profile by means of Eq. (2).

**Fig. 9:**
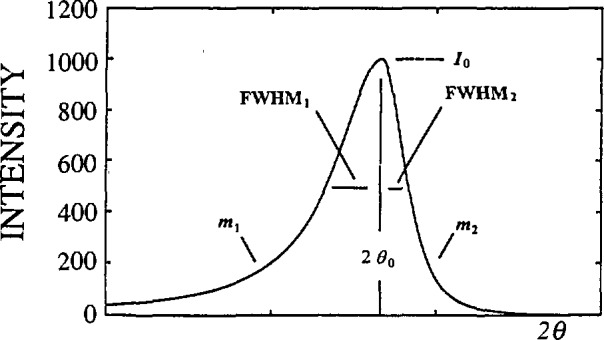
A split-Pearson VII profile. The two half profiles have same peak position and intensity. Adapted from Howard and Preston [4].

In the program SHADOW, instrumental parameters are determined by fitting the split-Pearson VII function (see Fig. 9) to line profiles of a standard specimen:

$$I(x)=I(0)\frac{1}{{\left(1+\frac{x^{2}}{mc^{2}}\right)}^{m}},$$ (71)

with

$$c=\frac{\beta \Gamma (m)}{\sqrt{m\pi }\Gamma (m-1/2)}.$$ (72)

Here, *m* = 1 or *m* = ∞ yields a Cauchy or Gauss function, respectively. Refined full widths at half maximum (FWHM) and shape factors *m* for both low-angle and high-angle sides of the profiles are fitted with second-order polynomials. To fit the FWHM, we used Eq. (69), and for the shape factors

$$m(2\theta )=U^{\prime }{\left(2\theta \right)}^{2}+V^{\prime }(2\theta )+W^{\prime }.$$ (73)

The resulting coefficients *U, V, W, U*′, *V*′, and *W*′ permit synthesis of the asymmetrical instrumental line profile at any desired angle.

**Fig. 10:**
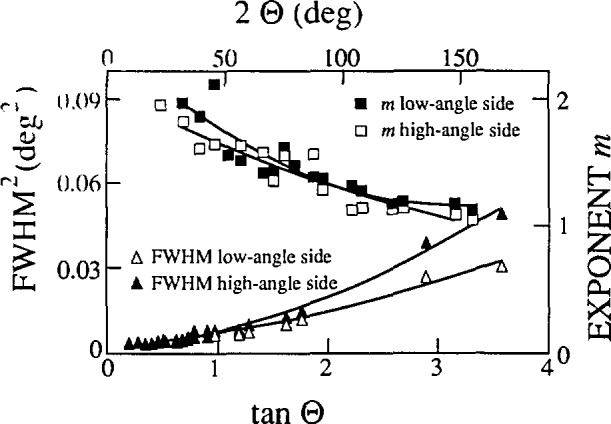
Refined FWHMs and shape factors (exponents) *m* for low-angle and high-angle sides of LaB_6_ line profiles. Second-order polynomials were fitted through points.

The basic requirement on the standard specimen is, however, to show as small a line broadening as possible. To minimize physical contributions to the Une broadening of the standard specimen, a few moments were emphasized as follows. Because diffraction-line width depends strongly on degree of annealing, it is preferable to use some reference powder-diffraction standard. Furthermore, asymmetry in the peak profiles is introduced by axial divergence of the beam, flat specimen surface, and specimen transparency [24]. Choosing a standard specimen with low absorption coefficient would cause transparency effects to dominate. If the studied specimen has a large absorption coefficient (compared to the standard), this might produce a fictitious size contribution and errors in microstrains. All of the specimens studied have absorption coefficients exceeding 1000 cm^−1^ so a NIST standard reference material 660 LaB_6_ was chosen to model the instrumental broadening (*μ* = 1098 cm^−1^). According to Fawcett et al. [130], LaB_6_ showed the narrowest lines of all studied compounds. Furthermore, LaB_6__has a primitive cubic structure (space group $Pm\bar{3}m$) resulting in relatively large number of peaks equally distributed over *2θ*. This allows for better characterization and lower errors of FWHMs and shape factors of split-Pearson VII functions (Fig. 10).

### 5.2 Applicability of the Method

To study the applicability of method described in Sec. 4, we first studied simple cubic-structure materials such as silver and tungsten. Tungsten has very narrow line profiles, allowing us to obtain the upper limit of domain sizes that can be studied. Silver is easily deformed, which provides a possibility to apply the method to broad line profiles. To test the case of relatively complicated patterns and weak line broadening, the method was also applied to La_1.85_Sr_0.15_CuO_4_ and La_2_CuO_4_ powders. In this section only the mechanical aspects of the line broadening are discussed. Discussion about the origins of broadening of superconductors can be found in Sec. 6.

#### 5.2.1 Silver and Tungsten Powders

**Fig. 11:**
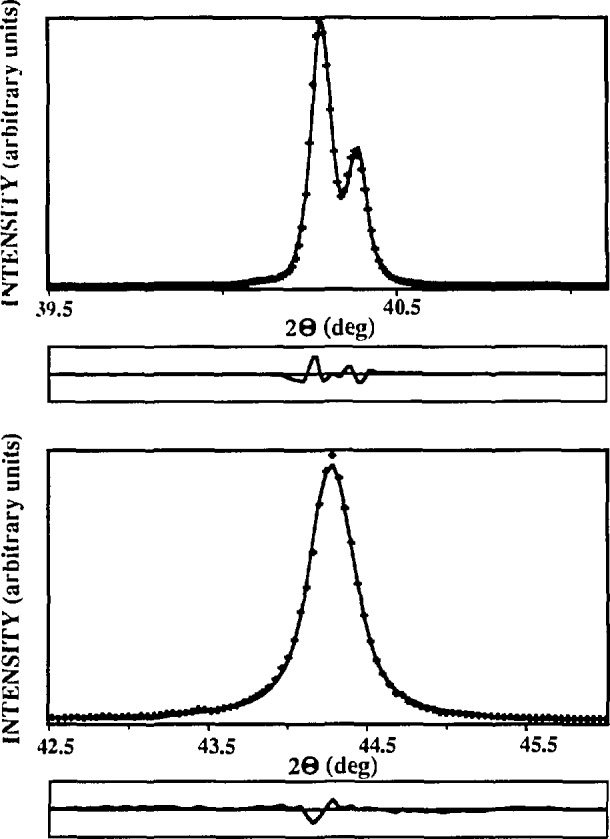
Observed points (pluses), refined pattern (full line), and difference pattern (below): (110) W untreated (upper); (200) Ag ground (lower).

**Fig. 12:**
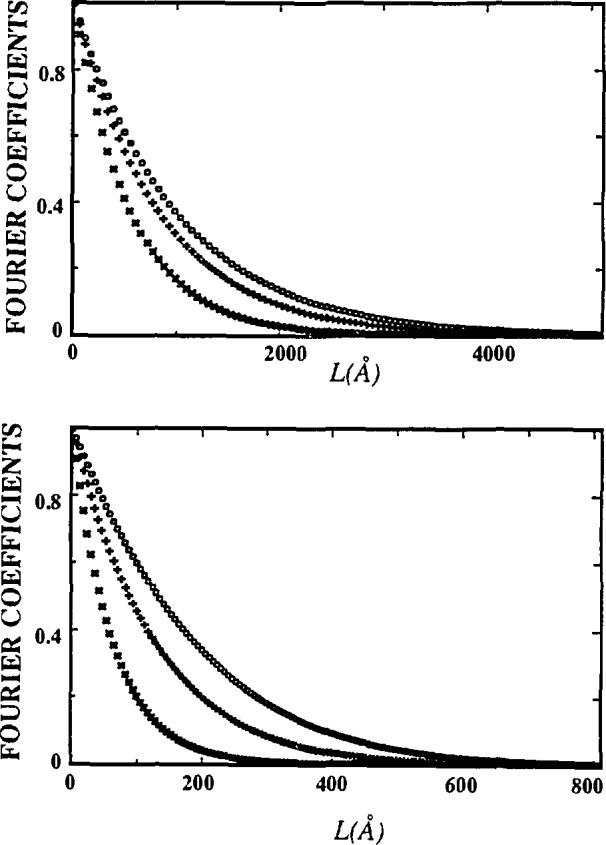
Fourier coefficients for the first- (pluses) and second-order (crosses) reflection, and size coefficients (circles): [111] Ag untreated (upper); [100] Ag ground (lower).

Figure 11 shows observed and refined peaks of tungsten untreated and silver ground powders. In Table 2 are listed results of fitted pure-specimen Voigt profiles for silver and tungsten specimens; Table 3 and Fig. 12 give results of the line-broadening analysis. Untreated tungsten powder shows relatively weak broadening. Instrumental profile FWHMs at angle positions of (110) and (220) tungsten lines are 0.059° and 0.081°, respectively, close to values measured for tungsten: 0.065° and 0.100°. Results in Table 3 reveal that small broadening is likely caused by domain sizes, because microstrains have negligible value. This pushes the limit for measurable domain sizes probably up to 4500–5000 A. However, one must be aware that weak specimen broadening implies higher uncertainty of all derived parameters. Moreover, the choice of the instrumental standard becomes more crucial.

**Fig. 13:**
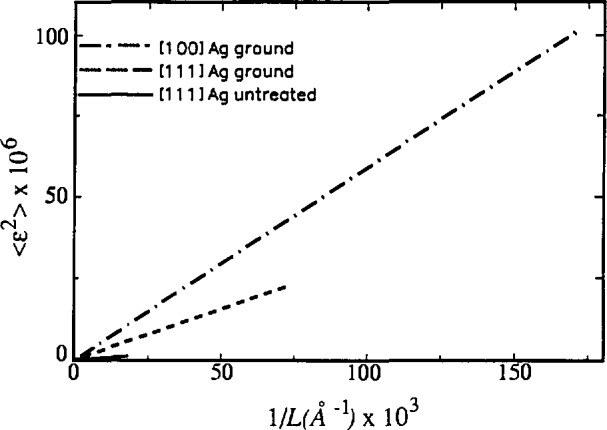
Mean-square strains 〈*ϵ*^2^〉 as a function of 1/*L*.

Both silver and tungsten line profiles become more Cauchy-like after grinding, which probably increases dislocation density in the crystallites. This is consistent with the presumption that small crystallites and incoherently diffracting domains separated by dislocations within grains affect the tails of the diffraction-line profiles [60,115]. Figure 13 illustrates the dependence of MSS on the reciprocal of the averaging distance *L*.

Errors in integral breadths allow estimation of errors in strain and size parameters (Sec. 4.5), but in some cases the refinement algorithm gives unreliable errors of integral breadths. When a particular parameter is close to the limiting value (for instance, 10^−5^ degrees has been put as the mini-mum value for integral breadths), errors become large. However, in Eqs. (63), (65), (66), (67), and (68), only the product of integral breadth and accompanying error is significant, which is roughly equal for Cauchy and Gauss parts. Errors of domain sizes and strains are of the same order of magnitude as errors of integral breadths.

The possible source of systematic error is potential inadequacy of Voigt function to accurately describe specimen broadening. This effect can not be evaluated analytically; but it would affect all derived parameters, especially the column-length distribution functions. The logical relationship between values of domain sizes (see Table 3) for different degrees of broadening indicates that possible systematic errors can not be large.

**Fig. 14:**
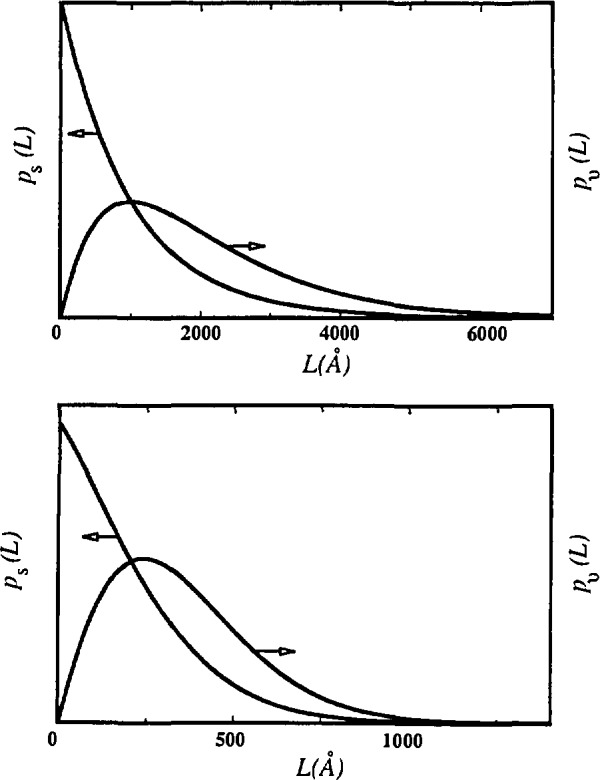
Surface-weighted and volume-weighted column-length distribution functions, normalized on unit area: [111] Ag untreated (upper); [100] Ag ground (lower).

**Fig. 15:**
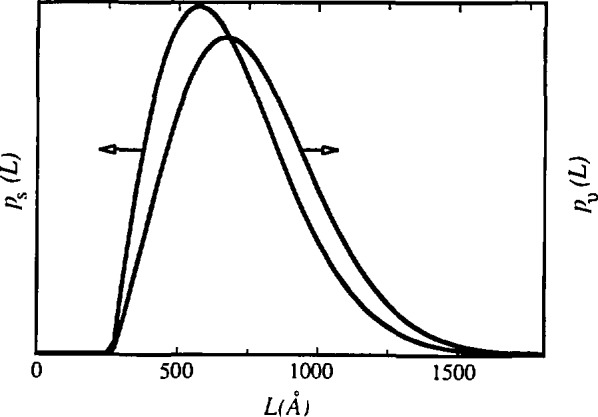
Surface-weighted and volume-weighted column-length distribution functions for [010] La_2_CuO_4_, normalized on unit area.

Equation (41) allows computation of volume-weighted and surface-weighted average domain sizes if respective column-length distribution functions can be obtained. Figure 14 gives surface-weighted and volume-weighted average column-length distribution functions following Eqs. (39) and (38). Width of the distribution function determines the relative difference between 〈*D*〉_s_ and 〈*D*〉_v_. The broader the distribution, the larger the differences, because small crystallites contribute more to the surface-weighted average. That is much more evident comparing the surface-weighted column-length distribution with the volume-weighted. If they have similar shape and maximum position, as in Fig. 15, differences are small. Conversely, if the surface-weighted distribution function has a sharp maximum toward smaller sizes, differences are larger (see Fig. 14). The difference between 〈*D*〉_s_ and 〈*D*〉_v_, and the actual mean dimension of the crystallites in a particular direction, also depends strongly on the average shape of the crystallites [52, 131, 132].

If experimental profiles are deconvoluted by the Stokes method, even for considerable specimen broadening, size coefficients *A*^s^ usually oscillate at larger *L* values [59, 106], preventing computation of the column-length distribution function. Few techniques were used to deal with this problem: successive convolution unfolding method [32, 133], smoothing, and iterative methods [134, 135, 136, 137]. Figures 14 and 15 show very smooth column-length distribution functions. However, they follow from size coefficients *A*^s^ that depend on the accuracy of the approximation for the distortion coefficient, given by Eq. (47). Equation (47) is exact if the strain distribution is Gaussian, but in general holds only for small harmonic numbers *n*, if strain broadening is not negligible.

#### 5.2.2 La_2-_*_x_*Sr*_x_*CuO_4_ Powders

**Fig. 16:**
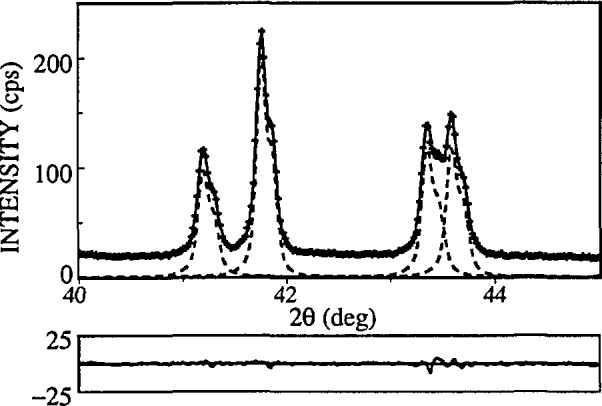
Observed points (pluses), refined pattern (full line), convoluted profiles (dashed line), and difference plot (below) for part of La_2_CuO_4_ pattern.

To test the applicability of the discussed method to more complicated patterns, two compounds with lower crystallographic symmetry were studied. La_1.85_Sr_0.15_CuO_4_ has a tetragonal K_2_NiF_4_-type structure, space group *I*4*/mmm*. La_2_CuO_4_ is orthorhombic at room temperature. Both compounds show slight line broadening and relatively highly overlap-ping peaks (see Fig. 16), which makes it very difficult, if not impossible, to perform a Stokes analysis.

Tables 4 and 5 list results from the fitting procedure and the analysis of specimen-broadened integral breadths. There are no qualitative differences in results for tungsten and silver powders. However, average errors are higher, as expected because of overlapping peaks, while weighted errors *R*_wp_ are surprisingly smaller. This fact illustrates the unreliability of *R*_wp_ when only a segment of the pattern is being refined, because it depends on the number of counts accumulated in points, as well as on the 2*θ* range of the refinement.

**Fig. 17:**
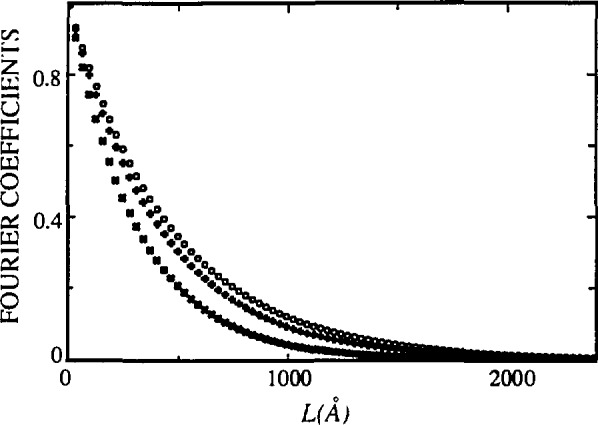
Fourier coefficients for the first- (pluses) and second-order (crosses) reflection, and size coefficients (circles) for [110] La_1.85_Sr_0.15_CuO_4_.

**Fig. 18:**
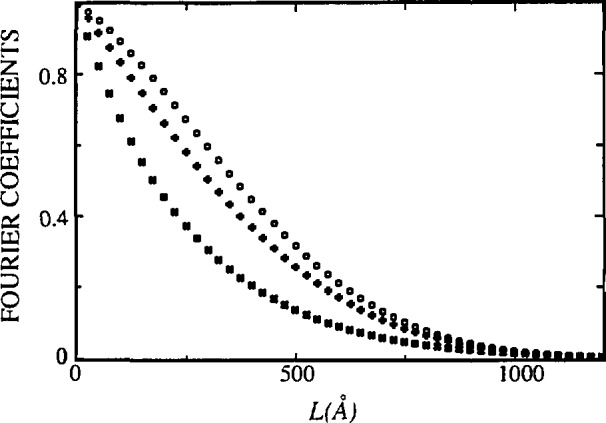
Fourier coefficients for first- (pluses) and second-order (crosses) reflection, and size coefficients (circles) for [010] La_2_CuO_4_.

Figures 17 and 18 represent Fourier coefficients of La_1.85_Sr_0.15_CuO_4_ [110] and La_2_CuO_4_ [010] directions. In the second plot, the *A*^S^(*L*)-versus-*L* plot shows a concave-downward part near *L* = 0, the so-called “hook” effect. It was shown in Sec. 4.2 that the “hook” effect originates because of underestimation of background, connected with the truncation of profiles. In the profile refinement, all peaks separated less than 4° 2*θ* have been included in the refinement region to avoid possible overlapping of peak tails, and specimen profiles have been truncated below 0.1 % of the maximum intensity. However, because the polynomial background was determined prior to profile refinement, it may be overestimated for complicated patterns containing many overlapping peaks. If background is refined with other profile parameters, undesirable correlation with integral breadths occurs.

### 5.3 Comparison with the Integral-Breadth Methods

Knowing the specimen integral breadths, simplified methods can be applied. In Sec. 2.1.2 we reviewed multiple-line and single-line integral-breadth methods. We shall compare the simplified multiple-line methods [Eqs. (23), (24), and (30)] and the single-line method [76] [Eqs. (29) and (30)] with results obtained for silver, tungsten, and La_2-_*_x_*Sr*_x_*CuO_4_ powders (Tables 3 and 5). We did not compare with the multiple-line Voigt-function method [26] because it was shown in Sec. 4.4 that it is incompatible with the Warren-Averbach analysis. In Table 6, integral breadth *β*_1_ was computed according to Eq. (11). This value was compared with *β*_A_, integral breadth computed from Fourier coefficients [58]:

$$\beta_{A}=\frac{1}{a_{3}\sum_{L=-\infty }^{\infty }A(L)}.$$ (74)

Results obtained by the Warren-Averbach analysis are significantly smaller for both size and strain. Integral-breadth methods give the upper limit for strain, so comparison is limited. Considering the approximation *e* = 1.25〈*ϵ*^2^(*L*)〉^1/2^ [138] with *L* = 〈D〉_v_/2 or L = 〈D〉_s_/2, strains resemble more closely than crystallite sizes. It must be noted, however, that this approximation makes sense only in the case of pure-Gauss strain broadening. Benedetti et al. [106] reported excellent agreement for both size and strain using Warren-Averbach and Cauchy-Gauss [Eq. (24)] methods. Results from Table 6, on the contrary, indicate that the Gauss-Gauss approximation gives values closest to the Warren-Averbach method for crystallite sizes, whereas the Cauchy-Gauss and especially the Cauchy-Cauchy approximation tend to give too large values. For strain, the trend is opposite. The Cauchy-Cauchy approximation resembles most closely a Warren-Averbach method. These results concur with the Klug and Alexander [24] comparison of the published size and strain values obtained by Warren-Averbach (Stokes) and integral-breadth methods. However, for x-ray diffraction, the Cauchy-Gauss assumption for the size-strain broadening is theoretically and experimentally more favored than the other two models. The fact that the Gauss-Gauss method for crystallite size and the Cauchy-Cauchy method for strain give more realistic results may mean that the presumption that size and strain broadening are exclusively of one type is an oversimplification.

The single-line method seems much less reliable. If Gaussian breadth is very small, no information about strain is obtained. Moreover, second-order reflections give much lower values of both size and strain than do basic reflections. However, if multiple reflections are not available, the single-line method can give satisfactory estimations.

### 5.4 Reliability of Profile Fitting

The profile fitting of a cluster of even severely overlapping peaks can be accomplished with a low error and excellent fit of total intensity. But how reliable is information obtained about separate peaks in the cluster? In the fitting procedure, it is possible to put constraints on the particular profile parameter to limit intensity, position, and width of the peak. If anisotropic broadening or different phases are present, constraints may not be realistic. Morever, acceptable results can be obtained even using a different number of profiles in refinement [4]. Hence, the first condition for the successful application of this method is knowledge of actual phases present in the sample and their crystalline structures.

**Fig. 19:**
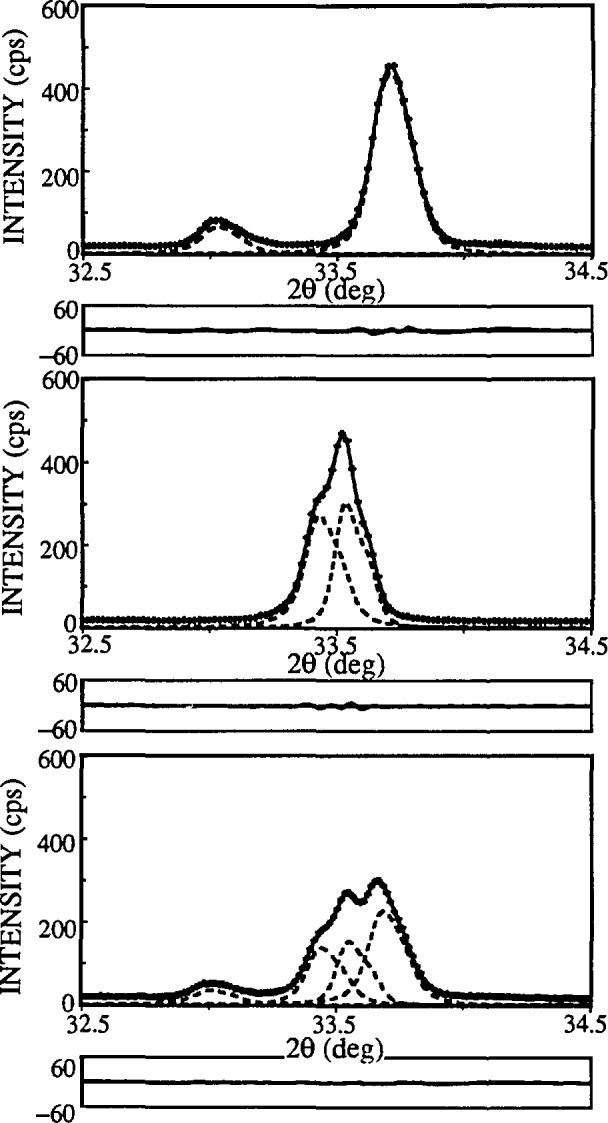
(upper) La_1.76_Sr_0.24_CuO_4_ (110) peak; (middle) La_1.94_Sr_0.06_CuO_4_ (020) and (200) peaks; (lower) (110), (020), and (200) peaks.

In this regard, two powders were measured: orthorhombic La_1.94_Sr_0.06_CuO_4_ has a slightly distorted K_2_NiF_4_-type structure (space group *Bmab, a* = 5.3510(2) Å, *b* = 5.3692 Å(2), *c* = 13.1931(7) Å) and tetragonal La_1.76_Sr_0.24_CuO_4_ (space group *I4/mmm a* = 3.7711(3) Å, *c* = 13.2580(8) Å). Data were collected for each specimen separately and for a specimen obtained by mixing the same powders. Because diffracted intensity of each phase was lower for the mixture, counting time was proportionally increased to obtain roughly the same counting statistics. Figure 19 shows two partially separated orthorhombic (020), (200) and (040), (400) peaks, overlapped with tetragonal (110) and (220) reflections. During the profile fitting of the mixed powders, (200) orthorhombic peak tended to “disappear” on account of neighboring (020) and (110) reflections. Its intensity was constrained to vary in range ± 30 % of (020) peak intensity, which can be justified if the crystallographic structure is known.

Table 7 contains results of fitted specimen-Voigt functions, as well as size and strain values obtained by the analysis of broadening. Comparing integral breadths of the starting and mixed powders, some profiles change from more Cauchy-like to Gauss-like and vice versa. This is especially evident for the (400) reflection, meaning that accurate determination of the profile tails is indeed affected in the cluster of overlapping peaks. However, integral breadth does not change significantly, so results of the multiple-line methods are not much influenced. On the contrary, the single-line method gives quite different results. This may support the conclusion of Suortti, Ahtee, and Unonius [43] that the Voigt function fails to account properly for size and strain broadening simultaneously if only Cauchy integral breadth determines crystallite size.

Table 7 shows that the results of Warren-Averbach analysis of starting and mixed powders agree in the range of standard deviations. However, errors are much larger, especially for the hidden (200)-(400) reflections. From Eq. (62) it follows that more counts (longer counting times) and more observables (smaller step-size) would lower standard deviations. Therefore, in the case of highly overlapping patterns, to obtain desirable accuracy, much longer measurements are needed, although that will not change the fact that higher overlapping implies intrinsically higher errors of all refinable parameters. To further minimize possible artifacts of the fitting procedure, it is desirable to include in the analysis as many reflections in the same Crystallographic direction as possible.

### 5.5 Remarks

By modeling the specimen size and strain broadening with the simple Voigt function, it is possible to obtain domain sizes and strains that agree with experiment and show a logical interrelationship. Furthermore, the Voigt function shows the correct 1/*Δ*(*2θ*)^2^ asymptotic behavior of peak tails, as expected from kinematical theory [139]. However, if the specimen profile is significantly asymmetric (because of twins and extrinsic stacking faults [140, 59]) or the ratio FWHM/*β* is not intermediate to the Cauchy (2/*π*) and Gauss (2(ln2/*π*)^1/2^) functions, then the Voigt function can not be applied [25]. Suortti, Ahtee, and Unonius [43] found good over-all agreement by fitting the Voigt function to the pure-specimen profiles of a Ni powder, deconvoluted by the instrumental function. However, more accurate comparison is limited because of unavoidable deconvolution ripples of specimen profiles. Furthermore, the Voigt function might not be flexible enough to model a wide range of specimen broadening, as well as the different causes of broadening. Therefore, the question whether the Voigt function can satisfactorily describe complex specimen broadening over the whole 2*θ* range in a general case needs further evaluation.

This method of Fourier analysis, with the assumed profile-shape function, is most useful in cases when the classical Stokes analysis fails; there-fore when peak overlapping occurs and specimen broadening is comparable to instrumental broadening. It was shown that for a high degree of peak overlapping, the fitting procedure can give unreliable results of Gauss and Cauchy integral breadths; that is, the peak can change easily from predominantly Cauchy-like to Gauss-like, and contrary. This can lead to illogical values of size-integral and strain-integral breadths, according to Eqs. (52), (53), (54), and (55), and to irregularities in the behavior of Fourier coefficients for large *L*. Simultaneously, if the size-broadened profile has a large Gauss-function contribution, the “hook” effect will occur. However, volume-weighted domain sizes 〈*D*〉_v_ and MSS 〈*ϵ*^2^(*L*)〉 for small *L* are much less affected, because they rely on both Cauchy and Gauss parts of the broadened profile. Any integral-breadth method that attempts to describe the size and strain broadening exclusively by a Cauchy or Gauss function, in this case, fails completely. For specimens with lower crystallographic symmetry. when many multiple-order reflections are available, but the peaks are overlapped, it is therefore desirable to include in the analysis as many reflections as possible, to minimize the potential artifacts of the fltting process.

## 6. Analysis of Superconductors

### 6.1 (La-M)_2_CuO_4_ Superconductors

Bednorz and Miiller [16] found the first high-temperature oxide superconductor in the La-Ba-Cu-O system. Soon after, substituting Ba with Sr and Ca, Kishio et al. [17] found superconductivity in two similar compounds. Later, many compounds with much higher transition temperatures (*T*_c_) were found. The (La-M)_2_CuO_4_ system remains favorable for study because the cation content, responsible for the superconductivity, is easier to control and measure than the oxygen stoichiometry. Also, this system provides a wide range of substitutional cation solubility in which the tetragonal K_2_NiF_4_-type structure is maintained. Compared with much-studied Y-Ba-Cu-O, this structure is simpler because it contains only one copper-ion site and two oxygen-ion sites.

The undoped compound La_2_CuO_4_ is orthorhombic at room temperature. It becomes superconducting either with cation substitution on La sites or increased oxygen content to more than four atoms per formula unit. Doping with divalent cations increases the itinerant hole carriers in the oxygen-derived electron band [141], which probably causes the superconductivity. However, different dopants influence the transition temperature *T*_c_. Although the unit-cell *c*-parameter simply reflects ionic dopant size [142], the in-plane unit-cell parameter (and accordingly the Cu-O bond length) correlates with *T*_c_ for different dopants and for different doping amounts [142, 143].

**Fig. 20:**
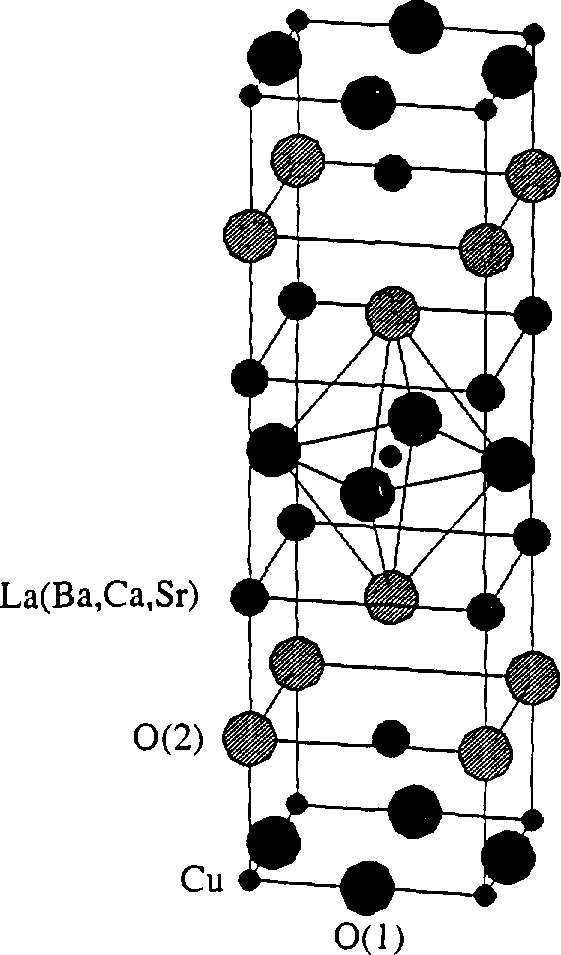
Crystal structure of (La-M)_2_CuO_4_ showing one unit cell.

La_1.85_M_0.15_CuO_4_ (M = Ba, Ca, Sr) have a tetragonal K_2_NiF_4_-type structure, space group *I*4/*mmm* with four atoms in the asymmetric unit [117]. One site is occupied with La, partially substituted with dopant ions (Fig. 20). La_2_CuO_4_ is orthorhombic at room temperature. The best way to correlate these two structures is to describe La_2_CuO_4_ in space group *Bmab;* the *c* axis remains the same, tetragonal [110] and $\left[1\bar{1}0\right]$ directions become [010] and [100] orthorhombic axes, and size of the unit cell is doubled [144].

**Fig. 21:**
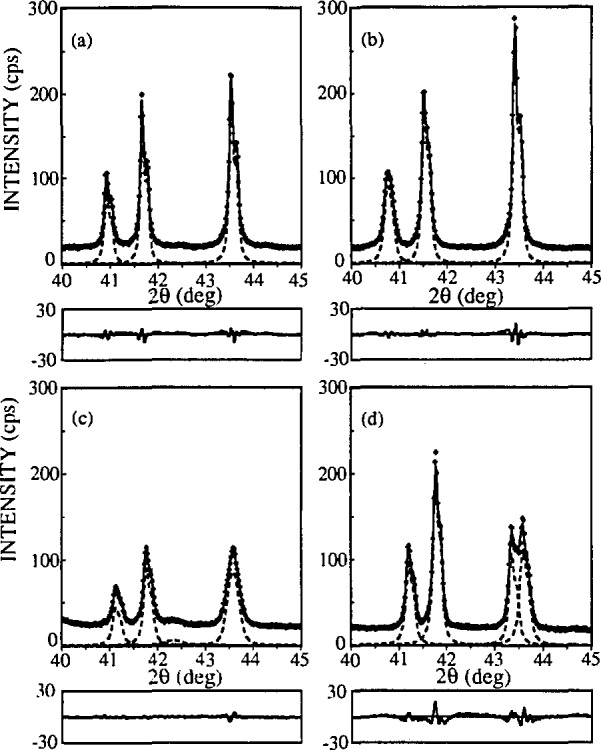
Diffraction patterns of La_1.85_M_0.15_CuO_4_ specimens: (a) M = Sr; (b) M = Ba; (c) M = Ca; (d) M = La.

Figure 21 shows parts of the x-ray diffraction patterns, observed and refined, of La_1.85_M_0.15_CuO_4_ (M = Sr, Ba, Ca) and La_2_CuO_4_. Refined lattice parameters are given in Table 8. Table 9 shows resuhs of the line-broadening analysis. Relatively weak peak broadening was observed, which can not be attributed to the proximity of the phase trans-formation; La_1.85_Sr_0.15_CuO_4_ is tetragonal down to = 170 K [145], La_1.85_Ba_0.15_CuO_4_ to 140 K [146,147], and La_1.85_Ca_0.15_CuO_4_ to 130 K [148]. Another possible reason for peak broadening might be continuous compositional variations in dopant content. The scanning Auger microprobe analysis of La_2-_*_x_*Sr*_x_*CuO_4_ compounds [149] showed that grain-boundary segregation of Sr is weak and limited to a narrow region near the grain surface. In the case of the separation of two phases with different Sr content and similar lattice parameters, asymmetry of some reflections would be observed [150]. Moreover, in the case of compositional inhomogeneity, microstrains 〈*ϵ*^2^(*L*)〉 would be roughly independent of the averaging distance *L* in the grains [151]; therefore distribution of strains would be Gaussian. For all specimens, microstrain behavior is identical; it decreases linearly with distance *L*. Rothman and Cohen [115] showed that such behavior of microstrains is caused by dislocations that might arise during grinding. Assuming the same applied stress, microstrain would be approximately inversely proportional to the elastic moduli of these materials. If mean microstrain is defined as the arithmetic average of strains for the [001] and [110] directions, results agree with values of the Young’s moduli for polycrystalline La_2_CuO_4_, La_1.85_M_0.15_CuO_4_ (M = Sr, Ba, Ca) [152]. However, other factors may contribute to the microstrain. Coordination-6 ionic radii of La^3+^, Sr^2+^, Ba^2+^, Ca^2+^ are 1.06, 1.16, 1.36, 1.00 Å, respectively [153]. Substitution of La^3+^ with ions of different sizes affects the lattice parameter [142] and may cause much larger strains in the [001] direction when some of the ions are replaced with Ba^2+^ than with Sr^2+^, in accord with results in Table 9. Substitution of Ca^2+^ ions produces an entirely different effect; microstrains in the [110] direction are larger. This may be explained by comparing effects of substitutional ions on the unit-cell *a*-parameter. The Ba-doped specimen has a larger *a*-parameter than the Sr-doped, but the Ca-doped *a*-parameter is largest, although Ca^2+^ has the smallest ionic size. This should increase the cell distortion in the *a-b* plane.

Table 9 reveals high anisotropy in these materials, consistent with other physical properties such as thermal expansion [154] and upper critical magnetic field [155]. Differences of domain sizes might arise from a layered structure in the [001] direction and consequently easy incorporation of stacking faults between regularly stacked layers. The stacking-fault and twin-fault probabilities can be estimated from the average surface-weighted domain sizes [59]:

$$\frac{1}{{{\langle D\rangle }_{s}}^{\left[hkl\right]}}=\frac{1}{\langle D\rangle }+c^{\left[hkl\right]}\text{fun}(\alpha ,\alpha^{\prime }).$$ (75)

Here, *c*^[^*^hkl^*^]^ is a constant for the particular [*hkl*] direction, and fun(*α*, *α′*) a linear combination of stacking-fault probability and twin-fault probability *α′*. The exact form of Eq. (75) was derived only for the cubic and hexagonal crystal structures. On condition that the “true” domain dimension *D* is isotropic or so large that the second term of Eq. (75) dominates, a linear function of stacking-fault and twin-fault probability is

$$\text{fun}(\alpha ,\alpha^{\prime })=c\left(\frac{1}{{{\langle D\rangle }_{s}}^{\left[110\right]}}-\frac{1}{{{\langle D\rangle }_{s}}^{\left[001\right]}}\right).$$ (76)

Here, *c* denotes a constant that depends only on geometrical factors and is the same for all three doped compounds.

**Fig. 22:**
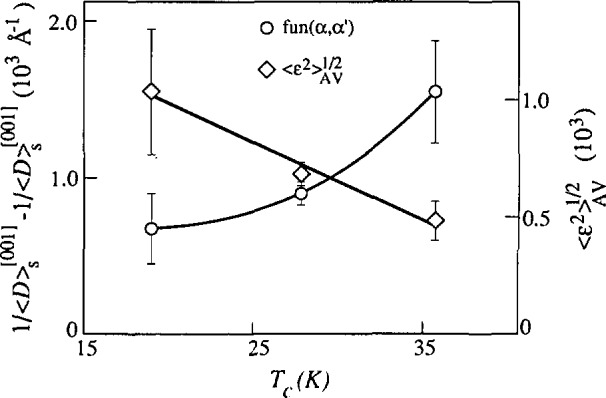
Arithmetic average of [110] and [001] root-meansquare strains and linear function of stacking-fault and twin-fault probabilities as a function of *T*_c_.

Figure 22 shows a possible correlation between *T*_c_ and fault-defect probabilities computed from. TEM observations of La_2-_*_x_*Sr*_x_*CuO_4_ revealed that stacking-fault density increased with Sr content up to *x* = 0.15 [156]. That study suggests that a shear mechanism forming the fault-boundary dislocations implies oxygen vacancies and/or cation deficiency. We did not measure oxygen content, but neutron-diffraction studies showed that oxygen deficiency starts to appear at much higher Sr content [157]. However, the increase of fault probability with the simultaneous decrease of microstrain in Fig. 22 supports the assumption that defects are introduced to accommodate lattice strains [156]. A similar increase in *T*_c_ was found in Y_2_Ba_4_Cu_8_O_20−_*_δ_* thin films [158]. Compounds with asymmetric broadened and shifted peaks showed higher *T*_c_, which was attributed to stacking faults. Although extrinsic stacking faults may cause asymmetric peak broadening [140], it is probably caused by twinning. Twinning in La-M-Cu-O materials is less abundant than in Y-Ba-Cu-O compounds, but it was observed in (La-Sr)_2_CuO_4_ monocrystals on (110) planes [159]. In the profile-fitting procedure, peak asymmetry is incorporated only in the instrumental function; therefore we could not model asymmetric peak broadening. However, any significant asymmetry of the specimen profile would cause an uneven fit of low-angle and high-angle sides of peaks, which was not observed. This gives much more importance to stacking faults in Eq. (76).

In Fig. 22, average microstrains, defined as the arithmetic average of [001] and [110] directions, correlate inversely with *T*_c_. However, we did not find a correlation of *T*_c_ and microstrains in a particular direction. [001] microstrains simply reflect difference in size of dopant and host La^3+^ ions (see Table 9).

In La_2_CuO_4_, (0*k*0) reflections are significantly broader than (*h*00). This has also been observed in low-temperature orthorhombic La_1.85_Sr_0.15_CuO_4_ and La_1.85_Ba_0.15_CuO_4_ [160], and explained by either stacking faults or possible lower symmetry. Our results reveal that domain sizes 〈*D*〉_s_ have identical values for [010] and [100] directions (Table 9). From Eq. (76), it follows that stacking-fault probabilities in these directions have the same values. However, microstrains in the [010] direction are roughly twice as large as in the [100] direction, which is responsible for the different broadening. In the case of doped La_2_CuO_4_ compounds, different microstrains might be caused by oxygen vacancies ordered along one direction in the *a-b* plane, similar to effects found in YBa_2_Cu_3_O_7−_*_δ_*. However, undoped La_2_CuO_4_ is not expected to have significant oxygen deficiency. Other possibilities are lower Crystallographic symmetry and/or La vacancies, although they were not reported for this system, but La deficiency was found in an La_2_CoO_4_ compound [161].

We can conclude that lattice strains and incoherently diffracting domain sizes confirm high anisotropy in these materials. This simple approach, however, does not allow unambiguous identification of the strain’s origin. Probably, the strains originate from dislocations and from different sizes of host and dopant ions. Domain sizes of all com-pounds are larger in the *c*-direction than in the *a*-*b* plane. This indicates existence of stacking faults and twins. However, because estimated errors are relatively large and only three materials were studied, the possible connection between stacking faults and *T*_c_ needs further study.

### 6.2 Bi-Cu-O Superconductors

**Fig. 23:**
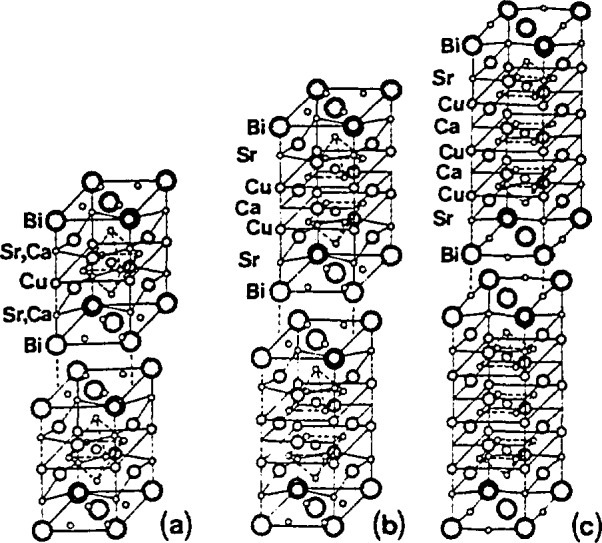
Average crystal structure of Bi_2_Sr_2_Ca*_m_*_−1_Cu*_m_*O_4+2_*_m_* for: (a) *m* = 1; (b) *m* = 2; (c) *m* = 3 [164].

Among the high-*T*_c_ superconductors, the. Bi-Cu-O compounds appear intriguing, especially because their incommensurate structure modulation remains incompletely understood [162, 163]. The crystal structure consists of perovskite-like SrO-(CuO_2_Ca)_m-1_-CuO_2_-SrO layers separated by NaCl-like BiO double layers (see Fig. 23 [164]). The number m of CuO_2_ layers alters the superconducting transition temperature *T*_c_ (for *m* = 1,2, and 3, *T*_c_ ≈ 10, 90, and 110 K, respectively). This two-dimensional layered structure makes these materials highly anisotropic and favours the creation of defects.

**Fig. 24:**
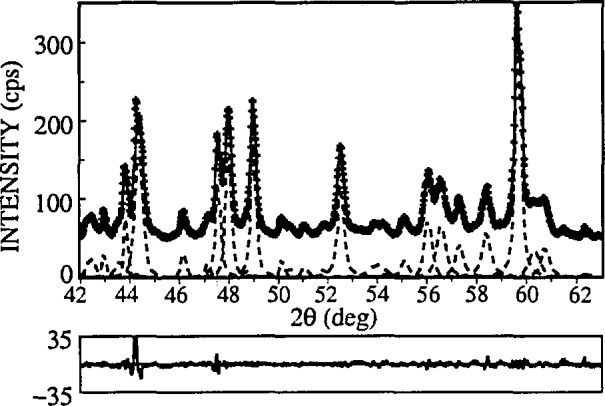
Part of Bi,Pb,Mg,Ba-2223 refined pattern. There are 48 fundamental reflections in this region.

Bulk sintered specimens showed relatively weak peak broadening. Moreover, the diffraction patterns contained many overlapping peaks because the compound crystallizes in an orthorhombic low-symmetry space *group A2aa* [165], and incommensurate modulation gives superlattice reflections. All patterns revealed a strong [001] texture (Fig. 24), making reflections other than (001) difficult to analyze. The lattice parameters vary depending on the exact compound stoichiometry and processing history, but characteristically they have almost identical *a* and *b* parameters, and large *c* parameter: *a* =5.4095 Å, *b* = 5.4202 Å, *c* = 30.9297 Å for Bi_2_Sr_2_CaCu_2_O_8_ (Bi-2212} [166] and *a* = 5.392 Å, *b* = 5.395 Å, *c* = 36.985 Å for (BiPb)_2_Sr_2_Ca_2_Cu_3_O_10_ (Bi,Pb-2223) [165]. Thus, we treated (*h*00) and (0*k*0) reflections as single peaks. Table 10 gives the results for three specimens: Bi-2212, (BiPb)_2_(SrMg)_2_(BaCa)_2_Cu_3_O_10_ (Bi,Pb,Mg,Ba-2223), which were one-phase specimens, and Bi,Pb-2223, containing approximately equal amounts of 2212 and 2223 phases. The in-plane lattice parameters of 2212 and 2223 phases differ little, hence only the [00*l*] directions of the mixed-phase specimen were evaluated.

Interpretation of the results in the case of these complicated compounds must be considered carefully. Microstrains are probably caused by dislocation arrays. However, substitution of different-sized ions and vacancies would also contribute to the strains, as would other kinds of disorder, especially stacking faults and twins. Table 10 shows larger strains in the [001] direction that in the basal plane. This can be explained by stacking disorder along the *c*-axis, which was frequently observed in electron-diffraction patterns as streaking [167, 168]. Easy incorporation of stacking faults is possible because of the two-dimensional weak-bonded layers (BiO double layers are spaced about 3 Å apart). The difference in microstrain between in-plane and the *c*-direction is much smaller for the Bi,Pb,Mg,Ba-2223 phase, probably because Pb lowers the faulting by stabilizing the higher-*T*_c_ 2223 phase [167]. For the Bi,Pb-2223 specimen, with two phases, there are two possibilities: Either the specimen consists mainly of a mix of two kinds of one-phase grains, or both phases occur within the same grains. The second possibility was confirmed experimentally [169, 170], and our results concur because microstrain in the 2212 phase is much smaller than in the one-phase Bi-2212 specimen. Processing conditions and cation content probably favour development of the macroscopic inter-growth of both phases within the grains; that is, microscopic defects grow to sufficiently large regions to give a diffraction pattern of another phase with the different number of CuO_2_ layers. As a result, strain is relieved, and both strains and domain sizes, being quite different in one-phase specimens, tend to equalize.

For the Bi-Cu-O specimens, the mean surface-weighted domain sizes are generally smaller than for (La-M)_2_CuO_4_ compounds. Therefore, the computation of the stacking- and twin-fault probabilities by using Eq. (75) may be wrong. It would be very instructive to compare the different broadening of satellite and fundamental reflections, which indicates a more pronounced stacking disorder in the modulated structure [171].

In summary, we found that for both Bi-2212 and Bi,Pb,Mg,Ba-2223 superconductors, the *c*-axis strain exceeds the in-plane strain. The Bi,Pb-2223 specimen, containing both 2212 and 2223 phases within the single grains, shows smaller incoherently diffracting domains and lower *c*-axis strain than the one-phase specimens. This fact may direct us to-ward the mechanism of the second-phase formation in these materials.

### 6.3 Remarks

It was shown that this particular method of the line-broadening analysis can give information about the size of incoherently diffracting domains and lattice strains in (La-M)_2_CuO_4_ and Bi-Clu-O superconductors. Moreover, results show high anisotropy of both size and strain parameters, as one may expect, because of the layered structure of all superconducting cuprates. Generally, structural line broadening is not large; that is a result of relatively large domain sizes (200–2000 Å) and small strains (0.02%–0.2%). It is interesting to compare these values with the published results for YBa_2_Cu_3_O_7−_*_δ_* (isotropic strains of 0.23% [77] and 0.05%–0.3% [82]). Although the different materials were studied, they seem too large, especially because they are the average of strains in different directions.

The main drawback of the line-broadening theory is the uncertainty about the origins of the line broadening; that is, it is difficult to distinguish the main effects responsible for the broadening. For the novel superconductors, particularly, the numerous defects (substitutions, vacancies, stacking faults, dislocations, and similar) can occur simultaneously, which makes it very difficult to determine their individual contributions. Furthermore, the crystal structures of most novel superconductors are relatively complicated. The average structure has mostly a slightly distorted tetragonal symmetry; very similar *a* and *b* lattice parameters, and a large *c* parameter, but *c* ≈ 36 for YBa_2_Cu_3_O_7−_*_δ_*, for instance. This results in a large number of overlapping peaks, and consequently in large errors of all results. This showed especially in the case of Bi-Cu-O superconductors (see Table 10). Following the discussion of reliability of profile fitting in Sec. 5.4, it would be desirable to include more than two reflections in the analysis of broadening, but higher-order peaks are usually too weak and overlapped. One way to partially solve this problem would be a whole pattern fitting [172, 173] and Rietveld structure refinement [2,3] in terms of size and strain parameters. This requires an accurate preset function to model specimen broadening and precise definition of angular dependence of size and strain parameters. The Rietveld programs that model the line broadening in terms of size and strain parameters are based either on the simplified integral-breadth methods [78, 95] or on the single-line method [174].

## 7. Conclusions

A method for analyzing the pure-specimen (structural) broadening of x-ray diffraction line profiles is proposed. By modeling the specimen size and strain broadenings with the simple Voigt function, it is possible to obtain domain sizes and strains that agree with experiment and show a logical interrelationship. Furthermore, some common consequences and problems in the Fourier-method analysis follow easily. The specimen function is convoluted with the instrumental function to match the observed x-ray diffraction-line profile. This avoids the Stokes deconvolution method, thus allowing analysis of patterns with highly overlapping peaks and weak structural broadening. Therefore, the method was applied to some novel high-*T*_c_ superconductors. We reached the following conclusions:

- Surface-weighted domain size depends only on the Cauchy integral breadth of the size-broadened profile.
- The “hook” effect occurs when the Cauchy-content of the size-broadened profile is underestimated, that is, for *β*_SC_〈(*π*/2)^1/2^ *β*_SG_. Usually this happens when the background is estimated too high, so the long tails of the Cauchy function are prematurely cut off.
- It also follows from conclusion (ii) that the volume-weighted domain size can not be more than twice the surface-weighted domain size (limiting value 〈*D*〉_v_ = 2〈*D*〉_s_ is obtained for the pure-Cauchy size-broadened profile). Minimum value for 〈*D*〉_v_/〈*D*〉_s_ should not be less than ~1.31.
- If the distortion coefficient is approximated with a harmonic term, this leads exactly to the Warren-Averbach method of separation of size and strain broadenings.
- In that case, mean-square strains decrease linearly with the distance *L*.
- It is possible to evaluate local strain (〈ϵ^2^(0)〉^1/2^) only in the case of pure-Gauss strain broadening. Then, root-mean-square strain is independent of the distance *L* and related to the upper limit of strain obtained from the integral breadth of the strain-broadened profile.
- If strain broadening is not described exclusively with the Gauss function, the multiple-line Voigt integral breadth and Warren-Averbach analyses can not give the same results for volume-weighted domain size, although 〈*D*〉_v_ is defined identically in both approaches, because the “apparent strain” *η = β*^D^(*2θ*)cot*θ* is not angular-independent.
- Smooth size coefficients give column-length distribution functions without oscillations and make possible a computation of volume-weighted average domain sizes up to ~5000 Å.
- Comparison with the simplified integral-breadth methods, in most cases, has shown not large, but systematic discrepancy of results, indicating that size and strain broadening can not be accurately modeled by a single Cauchy or Gauss function. Moreover, the application of the single-line method is much less reliable, and it should not be used where multiple reflections are available.
- The knowledge of all phases present in the sample and their crystallographic structure is required to successfully fit a cluster of overlapping peaks. Although it is very difficult to accurately determine the Cauchy-Gauss ratio of an overlapped peak, final results of domain size and strain are much less affected. Therefore, connecting the crystallite size exclusively with the Cauchy content and strain with the Gauss content of a specimen profile might be doubtful.
- Evaluated errors of domain sizes and strains increase substantially with the degree of peak overlapping. Hence, for complicated patterns, very accurate measurements are necessary and one needs to analyze as many reflections in the same crystallographic direction as possible.
- Line-broadening analysis of (La-M)_2_CuO_4_ and Bi-Cu-O compounds confirms high anisotropy in these materials.
- Strains in (La-M)_2_CuO_4_ probably originate from dislocations and from different sizes of host and dopant ions.
- Stacking-fault probability in (La-M)_2_CuO_4_ (M = Ba, Ca, Sr) increases with increasing *T*_c_, whereas the average strain decreases.
- In La_2_CuO_4_, different broadening of (*h*00) and (0*k*0) reflections is not caused by stacking faults.
- All Bi-Cu-O superconductors show larger strain in the *c*-direction than in the *a-b* plane, which can be explained by stacking disorder along the [001] direction.
- In the specimen with approximately equal amounts of Tl-2212 and Tl,Pb-2223 phases, it is likely that a secondary phase develops by the growth of the microscopic faulted regions of the primary phase.

## Figures and Tables

**Table 1:** Use of diffraction line-profile parameters

Position	Intensity	Shape	Shift	Method	Identification
√				Indexing	Cell parameters
√	√			Phase analysis	Identification and quantity
			√	Peak-shift analysis	Internal strain (residual stress)
√		√		Profile analysis	Microstrain, crystallite size, lattice defects
√	√	√		Structure refinement	Atomic positions, Debye-Waller factors, others

**Table 2:** Parameters of the pure-specimen Voigt function, as obtained from profile-fitting procedure for tungsten and silver powders

Specimen	*hkl*	2*θ*_0_(°)	*β*_C_(°)	*β*_G_(°)	FWHM(°)	R_wp_ (%)
W untreated	110	40.28	0.0065(7)	0.020(2)	0.021	9.0
	220	87.05	0.0301(6)	< 10^−5^	0.019	4.4
W ground	110	40.29	0.100(2)	0.038(4)	0.080	9.0
	220	87.05	0.222(3)	0.069(6)	0.166	4.2
Ag untreated	111	38.06	0.056(1)	< 10^−5^	0.036	10.5
	222	81.51	0.105(4)	0.0008(21)	0.067	9.7
	200	44.25	0.118(2)	< 10^−5^	0.075	10.5
	400	97.86	0.274(21)	< 10^−5^	0.175	12.2
Ag ground	111	38.07	0.175(3)	0.038(12)	0.121	9.9
	222	81.51	0.410(26)	< 10^−5^	0.261	14.5
	200	44.24	0.365(10)	0.076(29)	0.250	9.9
	400	97.83	1.079(82)	< 10^−5^	0.687	14.5

**Table 3:** Microstructural parameters for tungsten and silver powders

Specimen	Direction	〈D〉_s_(Å)	〈D〉_v_(Å)	〈*ϵ*^2^(*a*_3_)〉^1/2^	〈*ϵ*^2^(〈D〉_v_/2)〉^1/2^
W untreated	[110]	3200(200)	3500(200)	0.00038	0.00008(2)
W ground	[110]	620(20)	1030(30)	0.0023	0.00054(2)
Ag untreated	[111]	1000(20)	2000(20)	0.0010	0.00024(1)
	[100]	510(20)	1030(20)	0.0022	0.00047(5)
Ag ground	[111]	380(20)	650(20)	0.0047	0.00095(7)
	[100]	210(10)	350(20)	0.0100	0.00180(14)

**Table 4:** Parameters of the pure-specimen Voigt function, as obtained from profile-fitting procedure for La_2-_*_x_*Sr*_x_*CuO_4_ powders

Specimen	*hkl*	2*θ*_0_(°)	*β*_C_(°)	*β*_G_(°)	FWHM(°)	R_wp_ (%)
La_1.85_Sr_0.15_CuO_4_	004	26.95	0.046(4)	0.024(8)	0.042	3.4
	006	40.92	0.083(5)	< 10^−5^	0.053	6.2
	110	33.52	0.110(5)	< 10^−5^	0.070	12.5
	220	70.46	0.171(9)	< 10^−5^	0.109	4.3
La_2_CuO_4_	004	27.13	0.061(8)	0.045(9)	0.067	3.9
	006	41.19	0.124(7)	< 10^−5^	0.079	7.7
	020	33.16	0.074(10)	0.078(10)	0.102	2.9
	040	69.57	0.210(30)	0.026(81)	0.135	2.9
	200	33.45	0.074(8)	0.061(10)	0.087	6.9
	400	70.28	0.130(10)	0.023(95)	0.089	2.9

**Table 5:** Microstructural parameters for La_1.85_Sr_0.15_CuO_4_ and La_2_CuO_4_ powders

Specimen	Direction	〈D〉_s_(Å)	〈D〉_v_(Å)	〈*ϵ*^2^(*a*_3_)〉^1/2^	〈*ϵ*^2^(〈D〉_v_/2)〉^1/2^
La_1.85_Sr_0.15_CuO_4_	[001]	1700(300)	2000(200)	0.0024	0.00049(9)
	[110]	470(20)	940(20)	0.0017	0.00044(6)
La_2_CuO_4_	[001]	1100(100)	1200(100)	0.0038	0.0008(1)
	[010]	680(50)	760(50)	0.0033	0.0007(2)
	[100]	680(50)	810(50)	0.0016	0.0003(3)

**Table 6:** Comparison of results obtained with the integral-breadth methods: Cauchy-Cauchy (C-C), Cauchy-Gauss (C-G), Gauss-Gauss (G-G), and single-line (S-L) analysis

Specimen	*hkl*	*β*_1_ × 10^3^ (Å^−1^)	*β*_A_ × 10^3^ (Å^−1^)	〈D〉_v_ (Å)	〈D〉_v_(Å)				〈*ϵ*^2^(〈D〉_v_/2)〉^1/2^ × 10^3^	*e* × 10^3^			
					C-C	C-G	G-G	S-L		C-C	C-G	G-G	S-L
W untreated	110	0.259	0.259	3500	3700	3810	3810	14500	0.08	−0.01	−0.03	−0.05	0.24
	220	0.247	0.247					4040					0^a^
W ground	110	1.25	1.25	1030	2240	1450	1220	940	0.54	0.90	0.93	1.05	0.45
	220	2.04	2.05					550					0.32
Ag untreated	111	0.600	0.600	2000	3340	2390	2190	1670	0.24	0.35	0.39	0.95	0^a^
	222	0.901	0.900					1110					0^a^
	200	1.24	1.24	1030	2270	1470	1230	810	0.47	0.82	0.85	0.95	0^a^
	400	2.04	2.04					490					0^a^
Ag ground	111	1.98	2.00	650	2080	1240	910	530	0.95	1.79	1.82	1.97	0.48
	222	3.52	3.52					280					0^a^
	200	4.03	4.06	350	9380	4780	1320	260	1.80	4.05	4.05	4.08	0.82
	400	8.03	8.03					120					0^a^
La_1.85_Sr_0.15_CuO_4_	004	0.657	0.650	2000	5230	3290	2660	1970	0.49	0.76	0.78	0.88	0.44
	006	0.881	0.880					1140					0^a^
	110	1.19	1.19	940	1240	1000	970	840	0.44	0.52	0.64	0.80	0^a^
	220	1.58	1.58					630					0^a^
La_2_Cu_4_	004	0.988	0.998	1200	2980	1940	1630	1490	0.8	1.07	1.12	1.27	0.81
	006	1.32	1.31					760					0^a^
	020	1.43	1.42	760	1200	910	860	1240	0.7	0.79	0.90	1.10	1.14
	040	1.98	2.00					510					0.16
	200	1.26	1.24	810	810	810	810	1250	0.3	0.02	0.11	0.15	0.89
	400	1?S	1.26					830					0.14

a Not possible to evaluate because of too small Gauss integral breadth.

**Table 7:** Comparison between two specimens run separately and mixed togetlier

Specimen	*hkl*	2*θ*_0_(°)	*β*_C_(°)	*β*_G_(°)	〈D〉_s_(Å)	〈D〉_v_(Å)	〈*ϵ*^2^(〈*D*〉_v_/2)〉^1/2^ × 10^3^
La_1.76_Sr_0.24_CuO_4_	110	33.69	0.058(6)	0.094(7)	780(60)	850(60)	1.0(1)
	220	70.73	0.22(2)	0.13(2)			
La_1.94_Sr_0.06_CuO_4_	020	33.42	0.084(9)	0.55(14)	500(90)	1000(200)	0.7(1)
	040	70.12	0.10(2)	0.14(1)			
	200	33.53	0.0216(85)	0.061(8)	1100(100)	1200(100)	0.3(1)
	400	70.40	0.026(11)	0.088(8)			
Mix	110	33.67	0.083(21)	0.064(14)	800(300)	1200(300)	0.8(3)
La_1.76_Sr_0.24_CuO_4_ + La_1.94_Sr_0.06_CuO_4_	220	70.72	0.19(7)	0.13(4)			
	020	33.43	0.066(35)	0.070(23)	800(300)	1000(300)	0.7(4)
	040	70.12	0.116(93)	0.13(5)			
	200	33.54	0.024(156)	0.071(61)	900(700)	1000(600)	0.4(12)
	400	70.41	0.11(2)	0.003(320)			

**Table 8:** Lattice parameters and *T*_c_ at zero resistivity (Ac method at 10 Ma current)

Specimen	*a* (Å)	*b* (Å)	*c* (Å)	*T*_c_ (K)
La_1.85_Sr_0.15_CuO_4_	3.77814(7)		13.231(2)	36
La_1.85_Ba_0.15_CuO_4_	3.7846(2)		13.288(4)	28
La_1.85_Ca_0.15_CuO_4_	3.7863(7)		13.17(2)	19
La_2_CuO_4_	5.3558(2)	5.4053(2)	13.1451(8)	

**Table 9:** Results of line-broadening analysis for (La-M)_2_Cu_4_ specimens

Specimen	*hkl*	*β*_C_(°)	*β*_G_(°)	〈D〉_s_(Å)	〈D〉_v_(Å)	〈*ϵ*^2^(〈D〉_v_/2)〉^1/2^
La_1.85_Sr_1.15_CuO_4_	004	0.046(4)	0.024(8)	1700(300)	2000(200)	0.00049(9)
	006	0.083(5)	< 10^−5^			
	110	0.110(5)	< 10^−5^	470(20)	940(20)	0.00044(6)
	220	0.171(9)	< 10^−5^			
La_1.85_Ba_0.15_CuO_4_	004	0.049(6)	0.076(6)	1100(200)	1100(100)	0.0011(2)
	006	0.110(7)	0.089(9)			
	110	0.087(4)	0.00042(6)	560(20)	1110(20)	0.00024(5)
	220	0.116(3)	< 10^−5^			
La_1.15_Ca_0.15_CuO_4_	004	0.120(13)	0.032(22)	440(70)	780(90)	0.0007(4)
	006	0.146(12)	0.033(27)			
	110	0.045(25)	0.200(19)	340(30)	360(20)	0.0013(2)
	220	0.343(22)	< 10^−5^			
La_2_CuO_4_	004	0.061(8)	0.045(9)	1100(100)	1200(100)	0.0008(1)
	006	0.124(7)	< 10^−5^			
	020	0.074(10)	0.078(10)	680(50)	760(50)	0.0007(2)
	040	0.210(30)	0.026(81)			
	200	0.074(8)	0.061(10)	680(50)	810(50)	0.0003(3)
	400	0.130(10)	0.023(95)			

**Table 10:** Results of line-btoadening analysis for Bi-Cu-O supreconductors

Specimen	Phase	*hkl*	*β*_C_(°)	*β*_G_(°)	〈D〉_s_(Å)	〈*ϵ*^2^(〈D〉_v_/2)〉^1/2^
Bi-2212	2212	008	0.163(8)	0.01(6)	420(80)	0.0021(6)
		00.12	0.22(3)	0.05(6)		
		020+200	0.104(4)	< 10^−5^	580(30)	0.0007(1)
		040+400	0.18(2)	0.0004(6300)		
Bi,Pb,Mg,Ba-2223	2223	00.10	0.097(1)	0.0002(1700)	770(60)	0.0012(1)
		00.20	0.21(2)	0.007(150)		
		020 + 200	0.12(27)	0.13(10)	380(430)	0.0009(71)
		040 + 400	0.15(95)	0.21(33)		
Bi,Pb-2223	2212	008	0.23(2)	0.002(350)	220(30)	0.0013(26)
		00.12	0.25(5)	0.001(1000)		
	2223	00.10	0.22(8)	0.092(67)	230(190)	0.0012(197)
		00.20	0.23(99)	0.18(47)		

## Glossary

*a, b, c, m, m′*: General constants
*U, V, W*: General constants
*U′, V′, W′*: General constants
*i, x, z*: General variables
*A*: Fourier coefficient
*a*_3_: Edge of orthorhombic cell, orthogonal to diffracting planes
*D*: Domain size orthogonal to diffracting planes
*d*: Interplanar spacing
*e*: Upper limit of strain
FWHM: Full width at half maximum of profile
*f, F*: Pure-specimen (physically) broadened profile and its Fourier transform
*g, G*: Instrumentally broadened profile and its Fourier transform
*h, H*: Observed broadened profile and its Fourier transform
*hkl*: Miller indices
*I*: Intensity
*J*_c_: Critical superconducting current density
*K*: Scherrer constant
*k*: *β*_C_*/*(*π*^1/2^ *β*_G_), characteristic integral-breadth ratio of a Voigt function
*L*: *Na*_3_, column length (distance in real space) orthogonal to diffracting planes
*l*: Order of reflection
MSS: Mean-square strains
*N*: Average number of cells per column
*n*: Harmonic number
*p*: Column-length distribution function
*R*: Relative error
RMSS: Root-mean-square strains
*s*: 2sin *θ/λ* = 1/*d*, variable in reciprocal space
*T*_c_: Critical superconducting transition temperature
*w*: Observation weight
*Z*: Displacement of two cells in a column
*α*: Stacking-fault probability
*α′*: Twin-fault probability
*β*: β (2*θ*)cos *θ*_0_/*λ*, integral breadth in units of *s* (Å^−1^)
*γ*: Geometrical-aberration profile
*δ*: Fraction of oxygen atoms missing per formula unit
〈*ϵ*^2^/*L*)〉: Mean-square strain, orthogonal to diffracting planes, averaged over the distance *L*
*η*: “Apparent strain”
*θ*: Bragg angle
*θ*_0_: Bragg angle of *Kα_1_* reflection maximum
*λ*: X-ray wavelength
*σ*: Span of profile in real space
*ω*: Wavelength-distribution profile

**Superscripts**
D: Denotes the distortion-related parameter
S: Denotes the size-related parameter

**Subscripts**
C: Denotes Cauchy component of Voigt function
D: Denotes distortion-related parameter
*f*: Denotes pure-specimen (physically) broadened profile
G: Denotes Gauss component of Voigt function
*g*: Denotes instrumentally broadened profile
*h*: Denotes observed broadened profile
*S*: Denotes size-related parameter
*s*: Denotes surface-weighted parameter
*v*: Denotes volume-weighted parameter
*wp*: Denotes weighted-residual error

**Operators**
*: Convolution: *g*(*x*)**f*(*x*) = ∫*_g_*(*z*)*f*(*x* − z)d*z*
